# The burden of HIV-related stigma on clinical and quality of life outcomes: results from a systematic literature review

**DOI:** 10.1080/21642850.2026.2672790

**Published:** 2026-07-28

**Authors:** Irina Kolobova, Natalya Danchenko, Ian Jacob, Dennis Rice, Shannon Jones, Emily Matthews, Patrice Carter, Sanjeev Kumar Bhopal, Michelle Moorhouse

**Affiliations:** a ViiV Healthcare, Durham, NC, USA; b ViiV Healthcare, Rueil Malmaison, France; c ViiV Healthcare, London, UK; d Health Economics & Outcomes Research Ltd, Cardiff, UK; e ViiV Healthcare, Johannesburg, South Africa

**Keywords:** Adherence, HIV-related stigma, mental health, quality of life, systematic literature review

## Abstract

**Background:**

HIV-related stigma adversely impacts people with HIV. As part of a systematic literature review (SLR) to evaluate 6 research questions on HIV-related stigma among people with HIV, data from studies examining its prevalence and association with clinical and quality of life (QoL) outcomes are presented.

**Methods:**

Searches were conducted in MEDLINE® and Embase® via the OVID platform from May 2020 to May 2023 for prevalence and from database inception to May 2023 for the other 5 research questions. Relevant conferences, SLR bibliographies, and websites were searched from January 2021 to June 2023. Records were independently screened by 2 reviewers until ≥90% inter-rater reliability was achieved. Data were extracted by 1 reviewer and validated by another.

**Results:**

The reported prevalence of HIV-related stigma was broad across multiple stigma types and geographic regions, ranging from 11% to 94%. Most identified studies reported significant associations between higher HIV-related stigma levels and reduced HIV-specific healthcare engagement and retention (8/11), suboptimal antiretroviral therapy adherence (16/20), unsuppressed viral load (10/14), high levels of substance use (3/4), and suboptimal mental health (28/30). Most identified studies reported significant associations between higher HIV-related stigma levels and poorer QoL (23/27).

**Conclusions:**

The substantial evidence identified by this SLR suggests that HIV-related stigma is a significant challenge for people with HIV and negatively impacts QoL, mental health, and clinical outcomes. These findings underscore the importance of integrating stigma considerations into the dialogue between physicians and people with HIV to foster shared decision-making for treatment and support.

## Introduction

Advancements in antiretroviral therapy (ART) have improved prognoses among people with HIV (UNAIDS, 2018; Vitoria et al., 2019); however, people with HIV still experience discrimination and HIV-related stigma (Hedge et al., 2021). HIV-related stigma is the negative attitudes and beliefs about people with HIV, whereas discrimination is the behaviour that stems from those attitudes and beliefs (Centers for Disease Control Prevention, 2024). HIV-related stigma is recognised as a global public health concern, and the Joint United Nations Programme on HIV and AIDS (UNAIDS) has established 10-10-10 targets for 2025 to remove social and legal obstacles that limit access to and utilisation of HIV services, with the aim that ‘less than 10% of people living with HIV and key populations experience stigma and discrimination’ (UNAIDS, 2020). However, across geographies and populations with HIV, HIV-related stigma remains unacceptably high (UNAIDS, 2020). A recent meta-analysis of 40 studies in 13 countries reported an HIV-related stigma prevalence range of 19% to 88%, with 44% (69,978/171,627) of people with HIV reporting high stigma levels (Dessie & Zewotir, 2024).

**Figure 1.**
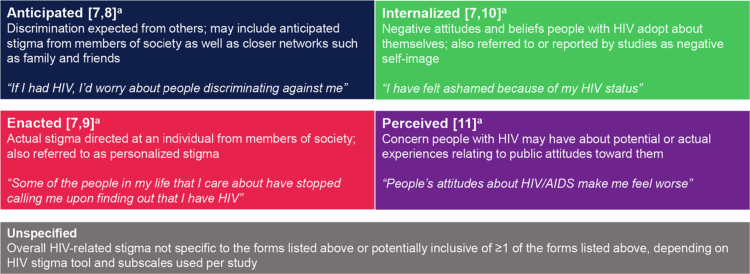
Forms of HIV-related stigma. ^a^Definitions may be author-dependent, and terms may be interchangeable or partially overlap within publications (Earnshaw & Chaudoir, 2009; Liu et al., 2020; United States Agency for International Development, 2006; UNAIDS; Alemu et al., 2022)

There are multiple forms of HIV-related stigma (Figure 1) (Alemu et al., 2022; Earnshaw & Chaudoir, 2009; Liu et al., 2020; UNAIDS, n.d.; United States Agency for International Development 2006), all of which can impact people with HIV across multiple aspects of their lives (Charles et al., 2012; Tran et al., 2019). HIV-related stigma can affect the full HIV care continuum from testing, treatment initiation, and adherence, to maintaining viral suppression in people with HIV (Stutterheim et al., 2022). Among people with HIV in Zambia and the Western Cape of South Africa, individuals who reported enacted or internalised stigma were significantly more likely to be non-adherent to ART than those who did not (*N* = 2020; *P* ≤ 0.03) (Jones et al., 2020). Furthermore, a study conducted in the United States found that people with HIV (*N* = 80) were more likely to report enacted stigma if they had a viral load > 5000 versus ≤ 5000 copies/mL (odds ratio [OR; 95% CI], 2.02 [0.96, 4.29]; *P* = 0.05) (Amico et al., 2021).

HIV-related stigma is associated with feelings of shame, depression, anxiety, and lower quality of life (QoL) (Charles et al., 2012; Tran et al., 2019). Depression is the most common mental health disorder in people with HIV and is correlated with experiences of HIV-related stigma (Dave, 2024; Fuenmayor & Cournos, 2022). Earnshaw et al. found that internalised and enacted stigma were significantly associated with depressive symptoms, and that depressive symptoms were significantly associated with substance use (*P* < 0.01) in people with HIV in the United States (Earnshaw et al., 2020). Notably, substance and alcohol use can lead to worsened disease outcomes including poor QoL and disease progression (Dave, 2024). Among 3991 self-reported people with HIV, the odds (95% CI) of reporting problems in ≥1 health-related QoL domain were 2.10 (1.72, 2.56; *P* < 0.001) times higher if a participant experienced HIV-related stigma at least once, compared with never experiencing HIV-related stigma (Hall et al., 2024). In a systematic literature review (SLR) and meta-analysis of 64 articles, Rzeszutek et al reported a negative association between HIV-related stigma and subjective well-being (Pearson *r* [95% CI], −0.31 [−0.36, −0.26]) (Rzeszutek et al., 2021). In addition to associations with depression and lower QoL, HIV-related stigma and discrimination affect employment in people with HIV. Findings from the People Living with HIV Stigma Index study, which included data from 13 countries across 4 regions, revealed 13% to 100% of participants in Fiji and Timor-Leste, respectively, lost their jobs or sources of income as a result of HIV-related stigma and discrimination (Internal Labour Organisation, 2018). Together, these data highlight the complexity and negative consequences of HIV-related stigma.

Due in part to its multi-faceted nature, there is no consensus on how to measure HIV-related stigma, leading to a complex body of research and unclear implications for future mitigation strategies and policy-making. In an effort to identify up-to-date published evidence to inform the current HIV-related stigma landscape from a global perspective, this SLR reports results from 4 research questions related to the association of HIV-related stigma with clinical and QoL outcomes, including healthcare engagement, ART adherence, viral suppression, comorbid conditions, and substance use, among people with HIV.

## Materials and methods

### Research questions

This article presents findings from the following 4 research questions:

1. What patient-reported outcome measures (PROMs) are used to assess HIV-related stigma in randomised controlled trials (RCTs) and observational studies?
2. What is the prevalence of HIV-related stigma reported across international geographies?
3. What published evidence is available on the association between HIV-related stigma and:
  - Treatment concepts (e.g. adherence, retention in care)
  - Clinical outcomes (e.g. viral suppression, CD4+ cell count)
  - Mental health (e.g. depression)
  - Health-related quality of life (HRQoL)
  - Employment and education
4. What published evidence is available on the impact of HIV-related stigma on:
  - Medication use for comorbid conditions
  - Economic outcomes (e.g. healthcare resource utilisation)
  - Drug, substance, and alcohol use

### Search strategy and inclusion/exclusion criteria

This SLR was conducted in alignment with PRISMA guidelines (Page et al., 2021) and a detailed protocol was registered with PROSPERO (CRD42023423538) (Carter et al., n.d.). Searches were conducted in MEDLINE® and Embase® via the OVID platform from May 2020 to May 2023 for the prevalence research question and from database inception to May 2023 for the other research questions. A restricted 3-year date range was applied to the prevalence search question to accurately capture the contemporary burden of HIV-related stigma. Conversely, searches for PROMs and clinical/economic associations were conducted from database inception to ensure a comprehensive capture of all foundational evidence. Conference proceedings and supplemental searches of European AIDS Treatment Group, National AIDS Trust, and European Centre of Disease Prevention and Control were conducted from 2021 to 2023. This search of conference proceedings and organisational websites were restricted to January 2021 to June 2023 to identify emerging, unpublished data and active guidelines while avoiding the duplication of older conference abstracts that have since been published as peer-reviewed manuscripts.

Search strategies were developed using a combination of indexed keywords and free text terms to identify relevant literature. For each search, specific medical subject headings and keyword terms were employed for HIV/AIDS, HIV-related stigma, and clinical and QoL outcomes (Supplemental Tables 1–4).

**Table 1.** Summary of studies reporting the prevalence of HIV-related stigma.

Publication	Population (*N*)	Tool used to measure HIV-related stigma	Type of HIV-related stigma assessed	Description of tool	Measure of prevalence	Reported prevalence (%)
Drumright et al. (2023), United States	People with HIV (6123)	NR	Internalised	4 items rated on a 5-point Likert scale (low (Vitoria et al., 2019) to high (UNAIDS, 2020))	Score of >2 with no change in score between 2 time periods (1 year apart from 2016-2022)	11
Pearson et al. (2021), United States	People with HIV (5968)	HIV Stigma Framework Scale (subscale)	Internalised	4 items rated on a 5-point Likert scale (strongly disagree (Vitoria et al., 2019) to strongly agree (UNAIDS, 2020))	Participants who agreed or strongly agreed with ≥1 item	29
Kota et al. (2023), United States	Transgender women with HIV (217)	MMP survey (3 items from the HIV stigma scale [negative self-image subscale])	Internalised	3 items rated on a 5-point Likert scale (strongly disagree (Vitoria et al., 2019) to strongly agree (UNAIDS, 2020))	Participants who somewhat agreed or strongly agreed with ≥1 item	27
		MMP survey (3 items from the HIV stigma scale [personalised stigma subscale])	Personalised			52
		MMP survey (4 items from the HIV stigma scale [disclosure concerns and concerns with public attitudes])	Anticipated	4 items rated on a 5-point Likert scale (strongly disagree (Vitoria et al., 2019) to strongly agree (UNAIDS, 2020))		Disclosure concerns: 87 Concerns with public attitudes: 69
		MMP survey (10 items from the HIV stigma scale [4 subscales: personalised stigma, disclosure concerns, negative self-image, concerns with public attitudes])	Unspecified	10 items rated on a 5-point Likert scale (strongly disagree (Vitoria et al., 2019) to strongly agree (UNAIDS, 2020))		94
Williams et al. (2022), United States	People with HIV (603)	HIV stigma scale (negative self-image subscale)	Internalised	3 items rated on a 5-point Likert scale (strongly disagree (Vitoria et al., 2019) to strongly agree (UNAIDS, 2020))^a^	Participants who reported higher HIV-related stigma levels	35
		HIV stigma scale (personalised stigma subscale)	Personalised			57
		HIV stigma scale (anticipated stigma subscale)	Anticipated	4 items rated on a 5-point Likert scale (strongly disagree (Vitoria et al., 2019) to strongly agree (UNAIDS, 2020))^a^		54
		HIV stigma scale (3 subscales: negative self-image, personalised stigma, anticipated stigma)	Unspecified	10 items rated on a 5-point Likert scale (strongly disagree (Vitoria et al., 2019) to strongly agree (UNAIDS, 2020))^a^		54
Noori et al. (2022), Europe	People with HIV (3272)	ECDC, EATG, and AAE developed stigma survey	Internalised	3 items rated on a 5-point Likert scale (strongly disagree (Vitoria et al., 2019) to strongly agree (UNAIDS, 2020))	Participants who agreed or strongly agreed with each stigma item	Range: 27–57
			Enacted	6 questions to assess experienced stigma from family and friends and recency of experience	Participants who responded positively to each item	Range: 10–24
Zhang et al. (2015), China	People with HIV (2987)	AIDS-related stigma scale	Internalised	8 items rated on a 4-point Likert scale (strongly disagree (Vitoria et al., 2019) to strongly agree (Prevention, 2024))	Participants who agreed or strongly agreed with ≥1 item	72
Algarin et al. (2020), United States	People with HIV (932)	Herek HIV-related stigma measure (abbreviated)	Enacted	10 items rated on a 4-point Likert scale	Participants who reported low/moderate or high enacted stigma levels	53
Maragh-Bass et al. (2021), United States	People with HIV (937)	Author-developed measure	Enacted	8 questions (yes/no) with an index range of 0–8^b^	Participants reporting 1–3 types of HIV stigma-related non-disclosure experience	Range: 17–33
Stutterheim et al. (2022), Europe	People with HIV (137–649)	Author-developed measure	Enacted	16 items rated on a 5-point Likert scale to measure 16 different settings (never (Vitoria et al., 2019) to very often (UNAIDS, 2020)); recoded to ‘never’ or ‘ever’	Participants who reported ever experiencing stigma in each of the 16 settings assessed	Range: 12–71
Wiginton et al. (2021), Europe	People with HIV (3475)	Positive voices survey (included 1 item from the HIV Stigma Index)	Enacted	1 question to assess enacted stigma in the healthcare setting (yes/no)	Participants who responded positively to the statement(s)	19
			Perceived			11
		Positive voices survey (included 2 items from the HIV Stigma Index)	Anticipated	2 questions to assess anticipated stigma in the healthcare setting (yes/no)		Range: 17–35
		Positive voices survey (included 4 items from the HIV Stigma Index)	Unspecified	4 questions to assess overall stigma in the healthcare setting (yes/no)	Participants who responded positively to ≥1 statement	40
Kall et al. (2020), Europe	Men with HIV (3110)	Positive voices survey (included 1 item from the HIV Stigma Index)	Enacted	1 question to assess enacted stigma in the healthcare setting in the past year (yes/no)	Participants who responded positively to experiencing stigma in the past year	7
	Women with HIV (1208)					9
	Transgender/non-binary people with HIV (39)					13
	Men with HIV (3110)	Positive voices survey (included 2 items from the HIV Stigma Index)	Anticipated	2 questions to assess anticipated stigma in the healthcare setting in the past year (yes/no)	Participants who responded positively to anticipating stigma in the past year	Range: 8–14
	Women with HIV (1208)					Range: 13–21
	Transgender/non-binary people with HIV (39)					Range: 13–15
	Men with HIV (3110)	Positive voices survey (included 1 item from the HIV Stigma Index)	Perceived	1 question to assess perceived stigma in the healthcare setting in the past year (yes/no)	Participants who responded positively to perceiving stigma in the past year	4
	Women with HIV (1208)					7
	Transgender/ non-binary people with HIV (39)					12
Brandelli Costa et al. (2022), Brazil	People with HIV (1786)	Stigma Index Brazil survey	Enacted	Multiple-answer question (7 different discriminatory experiences from health facility staff)	Participants who gave a positive response to any item	15
Kampouri et al. (2021), Europe	People with HIV (5563)	HIV stigma scale (disclosure concerns subscale)	Anticipated	12 items rated on a 4-point Likert scale	Participants who responded positively to any item	78
Beltran et al. (2020), United States	Men with HIV who have sex with men (NR)	Single statement as part of the NHBS survey	Perceived	Single statement used as an indicator of perceived stigma	Participants who agreed or strongly agreed with the single statement	2011: 19 2014: 16 2017: 23
Bayes-Marin et al. (2023), Europe	People with HIV (1060)	Neuro-QOL Item Bank v1.0-stigma	Perceived	2 questions used to assess self-perceived stigma in healthcare centres	Participants who responded positively to each statement	Range: 13–20

AAE; AIDS Action Europe; EATG, European AIDS Treatment Group; ECDC, European Centre for Disease Prevention and Control; MMP, Medical Monitoring Project; NHBS, National HIV Behavioural Surveillance; Neuro-QOL, Quality of Life in Neurological Disorders Item Bank v1.0 – Stigma; NR, not reported.

a A categorical measure was created based on the median score (higher stigma and lower stigma). ^b^Due to response distribution, categorical variables were assigned from 0–3 (0 = no stigma type, 1 = 1 stigma type, 2 = 2 stigma types, 3 = ≥3 stigma types).

**Table 2.** Summary of studies assessing the association of HIV-related stigma and initiation of HIV care or healthcare engagement and retention.

Publication	Study design	Form of HIV-related stigma	Outcome	Regression logistic (high vs low stigma levels), OR (95% CI)	*P* value (<0.05 considered statistically significant)
**Unadjusted analysis**					
Kerrigan et al. (2017), Brazil	Cross-sectional	Unspecified	Missed HIV care and treatment visits	1.43 (1.09, 1.89)	<0.05
Takada et al. (2020), United States	Cross-sectional	Unspecified	Engagement with HIV care (≥1 HIV primary care visit) 12 months before entering jail	0.79 (0.53, 1.19)	>0.05
Pearson et al. (2021), United States	Cross-sectional	Internalised	Attendance at next HIV primary care appointment after stigma assessment	0.89 (0.84, 0.95)	<0.0001
			Attendance at all HIV primary care appointments in next 12 months after stigma assessment	0.86 (0.82, 0.91)	<0.0001
Reif et al. (2019), United States	Cross-sectional	Internalised	Missed HIV care appointment in last 6 months	0.59 (0.14)^a^	<0.05
Petroll et al. (2023), United States	Cross-sectional	Perceived^b^	Lower engagement with HIV care	1.05 (NR)	0.84
		Perceived^c^		1.59 (NR)	<0.001
Wiginton et al. (2021), Europe	Cross-sectional	Unspecified (overall)	Reported unmet needs in peer support	1.37 (1.30, 1.44)^d^	NR
			Reported unmet needs in access to chronic health conditions management	1.43 (1.35, 1.50)^d^	NR
			Reported unmet needs in access to psychological care	1.44 (1.37, 1.52)^d^	NR
			Reported unmet needs in access to isolation help	1.45 (1.38, 1.53)^d^	NR
**Adjusted analysis**					
Christopoulos et al. (2019), United States	Cross-sectional	Internalised	Poor HIV care retention (≥2 missed primary care visits in prior year)	1.12 (1.05, 1.20)^e^	0.001
			Lack of 6-month primary care visit consistency (as part of HIV care)	1.09 (1.02, 1.17)^e^	0.008
Hussen et al. (2015), United States	Cross-sectional	Internalised	≥1 missed doctors’ appointments in last 3 months (as part of HIV care)	0.95 (0.91, 0.99)	0.03
Molina et al. (2018), United States	Cross-sectional	Enacted	Regular breast healthcare engagement (reporting clinical breast exam once in a year)	0.97 (0.93, 1.01)^f^	0.17
		Internalised		0.95 (0.91, 0.99)^f^	0.02
				**Logistic (low vs** **high stigma levels)**	
Yigit et al. (2020), United States	Prospective	Internalised	HIV visit adherence in the 48-week study period	2.17 (1.09, 4.35)^g^	0.03
				**Unstandardised linear (high vs low stigma levels),** ***β* value (SE)**	
Rice et al. (2017), United States	Cross-sectional	Internalised	Lower HIV visit adherence	–0.04 (0.02)	0.04
Petroll et al. (2023), United States	Cross-sectional	Perceived^b^	Low engagement in HIV care	0.05 (0.24)	NR
		Perceived^c^		0.46 (0.10)	NR

CNICS, Centre for AIDS Research (CFAR) Network of Integrated Clinical Systems; NR, not reported; OR, odds ratio.

a Standard error. ^b^33-item HIV stigma scale used to measure perceived stigma. ^c^HIV barriers to care scale (perceived stigma against people with HIV in their community: 1 item) used to measure perceived stigma. ^d^Unadjusted prevalence ratio (endorsing stigma vs not endorsing stigma). ^e^Adjusted for age, gender identity, sexual orientation, race/ethnicity, length of time in CNICS, and CNICS site. ^f^Adjusted for educational attainment and family history of breast cancer. ^g^Adjusted for age, race, gender, insurance status, and site.

**Table 3.** Summary of studies assessing the association of HIV-related stigma and HIV treatment adherence.

Publication	Study design	Form of HIV-related stigma	Outcome	Regression logistic (high vs low stigma levels), OR (95% CI)	*P* value (<0.05 considered statistically significant)
**Unadjusted analysis**					
Kerrigan et al. (2017), Brazil	Cross-sectional	Unspecified	ART adherence in last 4 days	0.60 (0.39, 0.92)	<0.05
Halkitis et al. (2014), United States	Cross-sectional	Unspecified	Missing ART doses in past 4 days	1.05 (1.00, 1.10)	<0.05
			Taking ART doses outside specified schedule in past 4 days	1.04 (1.01, 1.08)	<0.05
			Failing to follow ART dosing instructions	1.08 (1.03, 1.13)	<0.01
Algarin et al. (2020), United States	Retrospective	High enacted	ART non-adherence (<95%)	1.71 (1.08, 2.70)	0.02
		Moderate enacted		1.15 (0.82, 1.61)	0.43
		Healthcare-specific enacted		1.84 (1.15, 2.94)	0.01
Shrestha et al. (2019), United States	Cross-sectional	Enacted	ART adherence (≥95%)	0.78 (0.51, 1.20)	0.27
		Internalised		0.77 (0.54, 1.09)	0.14
		Anticipated		0.62 (0.39, 0.98)	0.04
Meyers-Pantele et al. (2022), United States	RCT	Personalised (enacted)	30-day ART adherence at 3-month follow-up	0.98 (0.96, 1.01)	>0.05
			30-day ART adherence at 6-month follow-up	0.97 (0.94, 0.99)	<0.01
Bogart et al. (2015), United States	Cross-sectional	Perceived alter^a^	ART adherence	0.36 (0.17, 0.76)	<0.01
Rudolph et al. (2022), United States	Cross-sectional	Internalised	ART adherence	0.64 (0.43, 0.95)^b^	NR
**Adjusted analysis**					
Seghatol-Eslami et al. (2017), United States	Cross-sectional	Internalised	ART adherence	0.61 (0.34, 1.08)^c^	0.09
Turan et al. (2016), United States	Cross-sectional	Internalised	ART adherence	0.76 (0.58, 0.99)^d^	0.042
				0.69 (0.52, 0.91)^d,e^	0.009
Blake Helms et al. (2017), United States	Cross-sectional	Internalised	HIV medication adherence	0.54 (0.31, 0.92)^f^	0.02
Turan et al. (2019), United States	Longitudinal	Internalised	ART adherence at ~2-year follow-up	0.61 (0.45, 0.82)^g^	0.001
Rice et al. (2019), United States	Cross-sectional	Anticipated stigma in healthcare setting	ART adherence	0.64 (NR)^h^	0.004
		Enacted stigma in healthcare setting		0.58 (NR)^i^	0.01
Shrestha et al. (2019), United States	Cross-sectional	Anticipated	ART adherence (≥95%)	0.38 (0.18, 0.83)	0.02
				**Logistic (low vs high stigma levels)**	
Yigit et al. (2020), United States	Prospective	Internalised	ART adherence	2.05 (1.06, 3.98)^j^	0.03
**Unstandardised analysis**				**Linear (high vs low stigma levels), *β* value (SE)**	
Stutterheim et al. (2022), Europe	Cross-sectional	Perceived	ART adherence	–0.50 (1.15)	NS
Seghatol-Eslami et al. (2017), United States	Cross-sectional	Internalised	ART adherence self-efficacy	–0.43 (NR)	0.005
Reif et al. (2019), United States	Cross-sectional	Internalised	HIV medication adherence	5.61 (1.91) [bivariate coefficient]	<0.05
				4.90 (2.13) [multivariate coefficient]	<0.05
		Enacted		2.23 (–3.35, –1.12)^k^ [multivariate coefficient]	<0.01
Rendina et al. (2019), United States	Cross-sectional	Unspecified	ART adherence	–0.36 (–1.29, 0.63)^k^	>0.05
**Standardised analysis**					
Stutterheim et al. (2022), Europe	Cross-sectional	Perceived	ART adherence	–0.04	NS
Camacho et al. (2020), United States	Cross-sectional	Anticipated	ART adherence	0.01 (–0.08, 0.08)^k^	0.91
Kalichman et al. (2022), United States	Cross-sectional	Anticipated	ART adherence	–0.12 (NR)	0.074
		Enacted		–0.11 (NR)	0.08
Meyers-Pantele et al. (2022), United States	RCT	Personalised (enacted)	7-day ART adherence at 3-month follow-up	–0.15 (–0.26, –0.03)^k^	<0.05
			7-day ART adherence at 6-month follow-up	0.02 (–0.10, 0.14)^k^	>0.05
Rendina et al. (2019), United States	Cross-sectional	Unspecified	ART adherence	–0.08 (NR)	>0.05
Takada et al. (2020), United States	Cross-sectional	Unspecified	ART adherence (30 days before incarceration)	–8.10 (–14.97, –1.23)^k^	<0.05
Mitzel et al. (2015), United States	Cross-sectional	Unspecified	Self-reported HIV medication adherence in past week	–0.34 (NR)	<0.01

ART, antiretroviral therapy; NR, not reported; NS, not significant; OR, odds ratio; RCT, randomised controlled trial; SES, socioeconomic status.

a Perceived alter stigma described as a person within a participant’s social network. ^b^Relative risk (95% CI). ^c^Adjusted by age, race, gender, SES, and ART duration. ^d^Adjusted by race, age, ART duration, injection and use of non-injectable drugs, income, and education. ^e^Reported for a subpopulation of women who identified as racial or ethnic minorities in a US cohort of women with HIV. ^f^Adjusted by age, racial identity, gender identity, SES, and ART duration. ^g^Adjusted by ethno-racial identity, age, ART duration, illicit drug use, income, education, and US region (south vs other). ^h^Adjusted by anticipated stigma from all other sources. ^i^Adjusted by enacted stigma from all other sources. ^j^Adjusted by age, race, gender, insurance status, and HIV clinical site. ^k^Reported 95% CI.

**Table 4.** Summary of studies assessing the association of HIV-related stigma and virologic suppression.

Publication	Study design	Form of HIV-related stigma	Outcome	Regression logistic (high vs low stigma levels), OR (95% CI)	*P* value (<0.05 considered statistically significant)
**Unadjusted analysis**					
Algarin et al. (2020), United States	Retrospective	High enacted	Unsuppressed VL (≥200 copies/mL)	1.19 (0.75, 1.89)	0.46
		Low/moderate enacted		0.96 (0.68, 1.34)	0.80
		Healthcare-specific enacted		1.08 (0.66, 1.80)	0.75
Takada et al. (2020), United States	Cross-sectional	Unspecified	Unsuppressed VL (<400 copies/mL) at first VL after incarceration	0.69 (0.51, 0.93)	<0.05
Amico et al. (2021), United States	RCT	Unspecified	High-risk vs low-risk viremia (>5000 vs ≤5000 copies/mL)	1.19 (0.99, 1.43)	0.06
		Anticipated		1.44 (0.88, 2.34)	0.14
		Enacted		2.02 (0.96, 4.29)	0.05
		Internalised		1.28 (0.94, 1.76)	0.12
Maragh-Bass et al. (2021), United States	Cross-sectional	Enacted (HIV disclosure stigma experiences)	Unsuppressed VL (>40 copies/mL)	Men: 0.77 (0.46, 1.28)^a^	>0.05
				Women: 1.63 (0.79, 3.35)^a^	>0.05
				Men: 1.57 (0.92, 2.68)^b^	<0.1
				Women: 1.64 (0.68, 3.95)^b^	>0.05
				Men: 0.87 (0.51, 1.48)^c^	>0.05
				Women: 2.06 (0.82, 5.18)^c^	>0.05
		Enacted (HIV diagnosis stigma experiences)		Men: 1.16 (0.79, 1.71)^d^	>0.05
				Women: 1.35 (0.58, 3.11)^d^	>0.05
Matheu et al. (2020), United States	Cross-sectional	Anticipated	Lack of virologic suppression (>20 copies/mL)	0.96 (0.92, 0.99)	0.02
**Adjusted analysis**					
Kay et al. (2018), United States	Cross-sectional	Enacted (community)	Suppressed VL (<200 copies/mL) vs not suppressed VL (≥200 copies/mL)	1.32 (0.51, 3.38)	0.57
		Enacted (healthcare setting)		3.23 (1.15, 9.06)	0.03
Christopoulos et al. (2019), United States	Cross-sectional	Internalised	Concurrent viremia (>200 copies/mL)	1.13 (1.03, 1.24)	0.01
Lipira et al. (2019), United States	Cross-sectional	Unspecified	VL suppressed (<200 copies/mL) versus not suppressed (≥200 copies/mL)	0.93 (0.89, 0.98)	<0.01
Kalichman et al. (2019), United States	Cross-sectional	Internalised	VL detectable (>100 copies/mL) versus undetectable (<100 copies/mL)	Men: 1.29 (0.89, 1.89)	>0.05
				Women: 1.67 (1.02, 2.74)	<0.05
		Enacted		Men: 0.99 (0.86, 1.13)	>0.05
				Women: 0.75 (0.58, 0.95)	<0.05
Christopoulos et al. (2020), United States	Longitudinal	Internalised	Unsuppressed VL (>200 copies/mL)	1.16 (1.05, 1.28)	0.004
				**Logistic (low vs high stigma levels)**	
Yigit et al. (2020), United States	Prospective	Internalised	VL suppressed (<200 copies/mL) versus not suppressed (≥200 copies/mL)	8.48 (1.83, 39.28)	0.006
Zhang et al. (2016), China	Cross-sectional	Perceived	VL high (>1000 copies/mL) versus low (1–49 copies/mL)	0.97 (0.92, 1.03)	>0.05
		Internalised		1.01 (0.97, 1.07)	>0.05
		Enacted		0.91 (0.60, 1.39)	>0.05
		Perceived	VL medium (50–1000 copies/mL) versus low (1–49 copies/mL)	1.05 (1.00, 1.09)	<0.05
		Internalised		1.05 (1.02, 1.09)	<0.0001
		Enacted		0.75 (0.51, 1.11)	>0.05
**Unstandardised analysis**				**Linear (high vs low stigma levels), *β* value (SE)**	
Rendina et al. (2019), United States	Cross-sectional	Unspecified	Undetectable VL (<200 copies/mL)	0.04 (-0.01, 0.08)^e^	>0.05
Kalichman et al. (2019), United States	Cross-sectional	Internalised	VL detectable (>100 copies/mL) versus undetectable (<100 copies/mL)	Total population: 0.41 (0.14)	NR
				Men: 0.26 (0.16)	NR
				Women: 0.55 (0.23)	NR
		Enacted		Total population: -0.13	NR
				Men: -0.01	NR
				Women: -0.26	NR
Miller et al. (2022), United States	Cross-sectional	Enacted (experiencing stigma at the pharmacy)	Lower likelihood of self-reported virologic suppression	2.09 (0.84)	0.013
**Standardised analysis**					
Rendina et al. (2019), United States	Cross-sectional	Unspecified	Undetectable VL (<200 copies/mL)	0.19	>0.05

NR, not reported; OR, odds ratio; RCT, randomised controlled trial; VL, viral load.

a 1 type of HIV disclosure stigma experience vs no experience of HIV disclosure stigma. ^b^2 types of HIV disclosure stigma experiences vs no experience of HIV disclosure stigma. ^c^≥3 types of HIV disclosure stigma experiences vs no experience of HIV disclosure stigma. ^d^Experience of HIV diagnosis stigma vs no experience of HIV diagnosis stigma. ^e^Reported 95% CI.

Eligibility criteria were established using 2 standard frameworks tailored to the nature of the specific research questions. These frameworks were not combined; rather, they were applied independently to distinct sets of research questions.

For questions focused on prevalence and observational data, we utilised the Condition, Context, and Population (CoCoPop) framework, which is the recommended methodological standard for systematic reviews of prevalence and incidence (Munn et al., 2015). The CoCoPop framework guided the eligibility criteria for the research questions regarding HIV-related stigma prevalence, PROMs, QoL, employment and education, clinical outcomes, mental health, and treatment concepts.

For questions evaluating specific impacts or comparative outcomes, we utilised the Population, Intervention, Comparison, Outcome, and Study Design (PICOS) framework. PICOS is a standardised tool widely used in evidence-based medicine to structure clinical research questions and determine literature search eligibility for comparative studies (Moher et al., 2015). The PICOS framework guided eligibility for the research questions examining the impact of HIV-related stigma on medication use for comorbid conditions, substance use (drug and alcohol), and healthcare resource utilisation costs.

Inclusion criteria for CoCoPop-assessed research questions included studies reporting HIV-related stigma, which encompassed social attitudes or perceptions toward HIV, fear of discrimination, and anxiety around sharing HIV status among adolescents (aged 12–18 years) and adults (aged >18 years) with HIV. Inclusion criteria for the PICOS-assessed research question included adolescents (aged 12–18 years) and adults (aged >18 years) with HIV. Only studies published in English were included.

### Record selection and data collection

Records were independently screened by 2 reviewers (EM and SJ) until ≥90% inter-rater reliability was achieved. Data were extracted by 1 reviewer and validated by another. Any discrepancies between the 2 reviewers were resolved by consensus or involvement of a third reviewer. Records for data extraction were processed by either a systematic or pragmatic approach, dependent on the research question(s) addressed by the study. A pragmatic approach limited to key outcomes was used for prevalence and PROMs, and a systematic approach was used for the other 2 research questions.

As the number of records identified by the SLR was large, the following additional selection criteria (in line with the protocol and agreed upon before data extraction) were applied to prioritise studies for data extraction and synthesis: only studies conducted in Brazil, China, Europe, and the United States; only studies published from 2013 to 2023; and only studies covering the research questions of interest. Additionally, when this SLR did not identify any studies reporting prevalence data in China for 2020 to 2023, an additional study published in 2015 (Zhang et al., 2015) that was identified via literature searches conducted for the other research questions was included. The Joanna Briggs Institute risk of bias tool was used according to study design (Briggs Institute, n.d.) and was conducted by a single reviewer and validated by a second.

### Data synthesis

The results are discussed narratively, as all statistical analyses discussed were conducted by the original study authors and *P* values were extracted verbatim.

### Ethics statement

This is a systematic literature reviewing previously generated data. Therefore, it was not necessary to seek ethical approval.

## Results

### Studies identified

**Figure 2.**
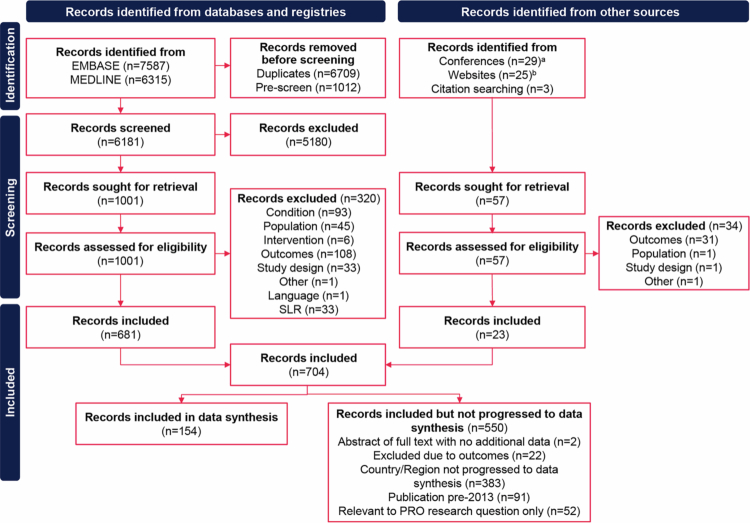
PRISMA flow diagram presenting study flow of this SLR. PRISMA, Preferred Reporting Items for Systematic Reviews and Meta-Analyses; SLR, systematic literature review. ^a^European AIDS Clinical Society Conference, International AIDS Society Conference on HIV Science, International AIDS Conference, Conference on Retroviruses and Opportunistic Infections, International Society for Quality of Life Research, International Association of Providers of AIDS Care, IDWeek, British HIV Association, Professional Society for Health Economics and Outcomes Research. ^b^European AIDS Treatment Group, National AIDS Trust, European Centre of Disease Prevention and Control.

The electronic database searches identified a total of 13,902 records (Figure 2). After removal of duplicates and exclusion of irrelevant records at pre-screening, 6181 titles and abstracts were screened against eligibility criteria. Full-text articles were sourced for 1001 records and eligibility criteria were reapplied, leaving 681 records meeting eligibility criteria for inclusion. A further 23 records were identified through supplementary searches. Following the additional refined eligibility criteria, a total of 154 records were included for synthesis.

### Patient-reported outcome measures of HIV-related stigma

**Figure 3.**
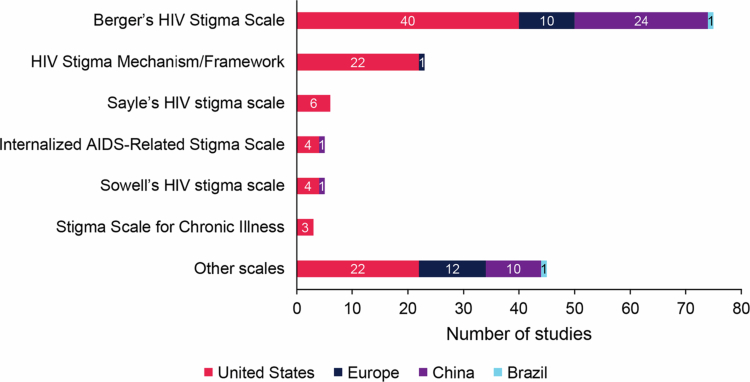
Distribution of patient-reported outcome measures employed in all included studies.

Patient-reported outcome measures for HIV-related stigma were employed in 148 studies, which used both validated (*n* = 26) and non-validated (*n* = 20) tools. Of the 148 studies, 98% (*n* = 145) were observational studies and 2% (*n* = 3) were RCTs. Validated measures were used in all 3 identified RCTs and 126/145 observational studies. Of the observational studies, 19 used non-validated measures, and 3 utilised both validated and non-validated measures. Among the observational studies and RCTs, the most commonly used PROM was the Berger HIV stigma scale (*n* = 75; Figure 3), with 14 studies reporting use of the full 40-item scale, 46 employing the abbreviated scale, and 15 using subscales. The HIV Stigma Framework was the second most commonly used PROM (*n* = 23). The greatest number of different measures were utilised to assess enacted (*n* = 22) HIV-related stigma, followed by internalised (*n* = 18) and perceived (*n* = 17) HIV-related stigma, suggesting more research interest in assessing these forms of HIV-related stigma.

### Prevalence of HIV-related stigma

This SLR identified 15 studies reporting prevalence data, including 7 studies conducted in the United States (Algarin et al., 2020; Beltran et al., 2020; Drumright et al., 2023; Kota et al., 2023; Maragh-Bass et al., 2021; Pearson et al., 2021; Williams et al., 2022), 6 in Europe (Bayes-Marin et al., 2023; Kall et al., 2020; Kampouri et al., 2021; Noori et al., 2022; Stutterheim et al., 2022; Wiginton et al., 2021), 1 in China (Zhang et al., 2015), and 1 in Brazil (Table 1) (Brandelli Costa et al., 2022). Across the 15 studies, 10 different tools were used to measure the prevalence of HIV-related stigma. In general, methodological procedures were well reported across the 15 included studies. However, in a few studies it was unclear whether HIV-related stigma had been measured in a valid and reliable way (eg, studies that used author-developed measures or single statements).

The reported prevalence of HIV-related stigma was broad across the multiple stigma types and geographic regions, with ranges of 11% to 94% in the United States (Algarin et al., 2020; Beltran et al., 2020; Drumright et al., 2023; Kota et al., 2023; Maragh-Bass et al., 2021; Pearson et al., 2021; Williams et al., 2022), 10% to 79% in Europe (Bayes-Marin et al., 2023; Kall et al., 2020; Kampouri et al., 2021; Noori et al., 2022; Stutterheim et al., 2022; Wiginton et al., 2021), and rates of 72% and 15% in China and Brazil, respectively (Brandelli Costa et al., 2022; Zhang et al., 2015). Overall, studies assessed the prevalence of enacted (*n* = 7), internalised (*n* = 6), anticipated (*n* = 5), perceived (*n* = 4), unspecified (*n* = 3), and personalised (*n* = 2) stigma. The single study conducted in China reported prevalence data for internalised HIV-related stigma only (72%) (Zhang et al., 2015); in the United States and Europe, the prevalence of internalised stigma was 11% to 35% (*n* = 4) (Drumright et al., 2023; Kota et al., 2023; Pearson et al., 2021; Williams et al., 2022) and 27% to 57% (*n* = 1) (Noori et al., 2022), respectively.

Although most studies focused on general populations of people with HIV, elevated HIV-related stigma levels were also observed in specific subgroups, such as transgender women, in whom stigma rates ranged from 27% to 94% (*n* = 1), and men who have sex with men, in whom perceived stigma levels in San Francisco rose from 16% in 2014 to 23% in 2017 (*n* = 1) (Beltran et al., 2020; Kota et al., 2023). Additionally, the prevalence of enacted stigma in healthcare settings in the Netherlands increased between 2007 (hospital, 26%; dentist, 29%; general practitioner [GP], 19%) and 2019/2020 (hospital, 34%; dentist, 34%; GP, 23%) (Stutterheim et al., 2022).

### Clinical burden of HIV-related stigma

#### HIV-related stigma and healthcare engagement and retention

**Figure 4.**
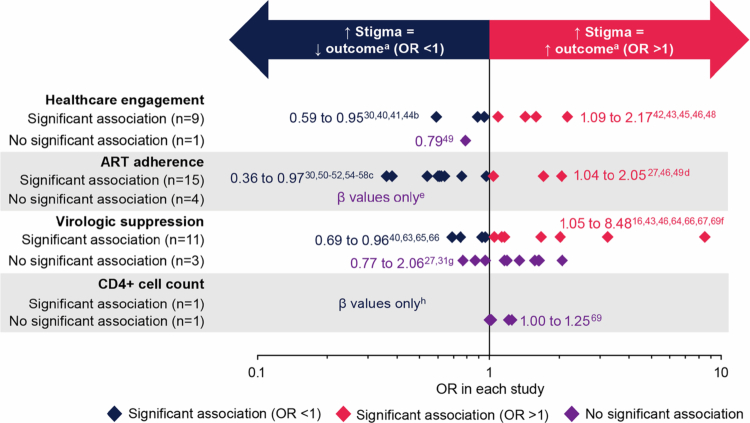
Clinical burden of HIV-related stigma. OR, odds ratio. ^a^Decreased outcomes (eg, attending HIV primary care appointments); increased outcomes (eg, missing HIV care and treatment visits). ^b^Includes *β* value −0.04 (Molina et al., 2018). ^c^Includes *β* value (range): −8.10 to −0.15 (Bogart et al., 2015; Camacho et al., 2020; Halkitis et al., 2014; Molina et al., 2018). ^d^Includes *β* value (range): 2.23 to 5.61 (Brandelli Costa et al., 2022). ^e^Includes *β* value (range): −0.50 to 0.01 (Stutterheim et al., 2022; Rendina et al., 2019; Rice et al., 2019; Turan et al., 2019). ^f^Includes *β* value 2.09 (Kalichman et al., 2019). ^g^Includes *β* value 0.04 (Turan et al., 2019). ^h^Includes *β* value −0.26 (Turan et al., 2019).

Eleven studies from the United States (*n* = 9) (Christopoulos et al., 2019; Hussen et al., 2015; Molina et al., 2018; Pearson et al., 2021; Petroll et al., 2023; Reif et al., 2019; Rice et al., 2017; Takada et al., 2020; Yigit et al., 2020), Europe (*n* = 1) (Wiginton et al., 2021), and Brazil (*n* = 1) (Kerrigan et al., 2017) evaluated the association of HIV-related stigma and healthcare engagement and retention, including HIV-specific healthcare engagement and retention (Figure 4; Table 2). Most studies used validated scales to measure HIV-related stigma, and one study created a survey based on the Healthcare Stigma/Discrimination scale from the 2015 HIV Stigma Index (Wiginton et al., 2021). Several outcomes were used to assess healthcare engagement and retention, including missed HIV care appointments in the last 6 months, poor HIV care retention (≥2 missed primary care visits), and ≥1 missed appointment in the last 3 months. Higher HIV-related stigma levels were significantly associated with reduced engagement and retention in general care (2/11 studies) (Molina et al., 2018; Wiginton et al., 2021) and HIV-specific healthcare (8/11 studies) (Christopoulos et al., 2019; Hussen et al., 2015; Kerrigan et al., 2017; Pearson et al., 2021; Petroll et al., 2023; Reif et al., 2019; Rice et al., 2017; Yigit et al., 2020).

The majority of studies (7/11) examined the association between higher internalised HIV-related stigma levels and retention and engagement in healthcare in the United States, with 6/7 focusing on HIV care specifically. Three studies reported statistically significant associations between higher internalised HIV-related stigma levels and poorer attendance at HIV primary care appointments (Christopoulos et al., 2019; Hussen et al., 2015; Pearson et al., 2021). Rice et al found that higher internalised stigma levels were significantly associated with lower HIV visit adherence (*β* value [SE], −0.04 [0.02]; *P =* 0.04) (Rice et al., 2017). In agreement with these findings, Reif et al reported higher internalised stigma levels were significantly associated with missing HIV medical appointments in the last 6 months (OR [SE], 0.59 [0.14]; *P* < 0.05) (Reif et al., 2019). Additionally, Yigit et al demonstrated that lower internalised HIV-related stigma levels were significantly associated with higher HIV visit adherence in a 48-week study period (OR [95% CI], 2.17 [1.09, 4.35]; *P* = 0.03) (Yigit et al., 2020).

#### HIV-related stigma and ART adherence and viral suppression

The association between HIV-related stigma and ART adherence was examined in 20 studies across the United States (*n* = 18) (Algarin et al., 2020; Blake Helms et al., 2017; Bogart et al., 2015; Camacho et al., 2020; Halkitis et al., 2014; Kalichman et al., 2022; Meyers-Pantele et al., 2022; Mitzel et al., 2015; Reif et al., 2019; Rendina et al., 2019; Rice et al., 2019; Rudolph et al., 2022; Seghatol-Eslami et al., 2017; Shrestha et al., 2019; Takada et al., 2020; Turan et al., 2016; Turan et al., 2019; Yigit et al., 2020), Europe (*n* = 1) (Stutterheim et al., 2022), and Brazil (*n* = 1; Figure 4; Table 3) (Kerrigan et al., 2017). Each of the studies reported measures of HIV-related stigma using validated scales. Outcomes for ART adherence varied between studies and included missing ART doses, taking doses outside of the specified schedule, ART non-adherence (<95%), and ART adherence (≥95%). Overall, higher HIV-related stigma levels were significantly associated with suboptimal ART adherence in 15/20 studies (Algarin et al., 2020; Blake Helms et al., 2017; Bogart et al., 2015; Halkitis et al., 2014; Kerrigan et al., 2017; Meyers-Pantele et al., 2022; Mitzel et al., 2015; Reif et al., 2019; Rice et al., 2019; Seghatol-Eslami et al., 2017; Shrestha et al., 2019; Takada et al., 2020; Turan et al., 2016; Turan et al., 2019; Yigit et al., 2020). Internalised stigma and its association with ART adherence was the most commonly assessed and was evaluated in 8 studies in the United States. Of these studies, 6 reported significantly poorer or suboptimal ART adherence in individuals reporting high internalised stigma levels (Blake Helms et al., 2017; Reif et al., 2019; Rudolph et al., 2022; Seghatol-Eslami et al., 2017; Turan et al., 2016; Turan et al., 2019). One study by Yigit et al reported a statistically significant association between lower internalised HIV-related stigma levels and increased likelihood of ART adherence (Yigit et al., 2020).

Fourteen studies reported data on the association between HIV-related stigma and viral suppression in the United States (*n* = 13) (Algarin et al., 2020; Amico et al., 2021; Christopoulos et al., 2019; Christopoulos et al., 2020; Kalichman et al., 2019; Kay et al., 2018; Lipira et al., 2019; Maragh-Bass et al., 2021; Matheu et al., 2020; Miller et al., 2022; Rendina et al., 2019; Takada et al., 2020; Yigit et al., 2020) and China (*n* = 1; Table 4) (Zhang et al., 2016). Generally, the identified studies used validated scales to measure HIV-related stigma, except for Maragh-Bass et al, which employed author-developed tools based on existing methods and literature (Maragh-Bass et al., 2021). Higher HIV-related stigma levels were significantly associated with a higher (or unsuppressed) viral load in 79% (*n* = 11) of studies (Amico et al., 2021; Christopoulos et al., 2019; Christopoulos et al., 2020; Kalichman et al., 2019; Kay et al., 2018; Lipira et al., 2019; Matheu et al., 2020; Miller et al., 2022; Takada et al., 2020; Yigit et al., 2020; Zhang et al., 2016). Researchers in a US study modelled individual- and community-level associations of viral load results with income inequality and stigma scores (Kalichman et al., 2019). Interestingly, the study found that women with detectable viral loads (>100 copies/mL) experienced significantly greater internalised stigma levels and significantly lower enacted stigma levels, but no significant associations were found with men’s viral loads.

#### HIV-related stigma and CD4+ cell count

The evidence identified on the association between HIV-related stigma and CD4+ cell count was limited and inconsistent (*n* = 2; Figure 4) (Rendina et al., 2019; Zhang et al., 2016), with a US study reporting a significant association between higher unspecified HIV-related stigma levels and lower CD4+ cell count in individuals aged ≥50 years (Rendina et al., 2019). Nonetheless, the study from China found no significant association between perceived, internalised, or enacted HIV-related stigma and CD4+ cell count (Zhang et al., 2016).

#### HIV-related stigma and prevalence of comorbid conditions

Thirty studies in the United States (*n* = 17) (Blake Helms et al., 2017; Brown et al., 2016; Chapman Lambert et al., 2020; Drumright et al., 2023; Felker-Kantor et al., 2019; Gonzalez et al., 2016; Hong et al., 2023; Hussen et al., 2022; Kay et al., 2018; Kreniske et al., 2022; Mitzel et al., 2015; Molina et al., 2018; Rendina et al., 2018; Thompson et al., 2022; Travaglini et al., 2018; Turan et al., 2022; Walsh et al., 2023), Europe (*n* = 3) (de Daas et al., 2019; Drewes et al., 2021; Murphy et al., 2018), and China (*n* = 10) (Gao et al., 2018; Greene et al., 2013; Li et al., 2016; Liu et al., 2018; Tao et al., 2017; Wang et al., 2018, 2022; Wu et al., 2015; Yang et al., 2020; Zhang et al., 2016) that reported data on the association between HIV-related stigma and comorbid conditions were identified. Validated scales used to measure HIV-related stigma were consistently implemented across the studies; however, details of the HIV stigma scale used in Li et al were absent (Li et al., 2016). Higher HIV-related stigma levels were significantly associated with depression (*n* = 15; Table 5) (Brown et al., 2016; Chapman Lambert et al., 2020; Felker-Kantor et al., 2019; Hong et al., 2023; Hussen et al., 2022; Kay et al., 2018; Li et al., 2016; Liu et al., 2018; Mitzel et al., 2015; Molina et al., 2018; Murphy et al., 2018; Rendina et al., 2018; Walsh et al., 2023; Yang et al., 2020; Zhang et al., 2016) and anxiety (*n* = 10) (Blake Helms et al., 2017; Brown et al., 2016; Felker-Kantor et al., 2019; Hong et al., 2023; Hussen et al., 2022; Li et al., 2016; Murphy et al., 2018; Rendina et al., 2018; Yang et al., 2020; Zhang et al., 2016), which was consistent across subpopulations and geographic regions. Higher HIV-related stigma levels were significantly associated with post-traumatic stress disorder (*n* = 3) (Gao et al., 2018; Gonzalez et al., 2016; Travaglini et al., 2018) and neurocognitive decline (*n* = 1) (Thompson et al., 2022). Additionally, individuals with higher HIV-related stigma levels were significantly more likely to have suicidal ideation (*n* = 4) (Kreniske et al., 2022; Wang et al., 2018, 2022; Wu et al., 2015).

**Table 5.** Summary of studies assessing the association of HIV-related stigma and depression.

Publication	Study design	Form of HIV-related stigma	Measure and outcome	Regression logistic (high vs low stigma levels), OR (95% CI)	*P* value (<0.05 considered statistically significant)
**Unadjusted analysis**					
Chapman Lambert et al. (2020), United States	Cross-sectional	Anticipated (family)	PHQ-8; higher score on the depression scale	1.42 (0.20, 1.69)	<0.05
		Anticipated (friends)		1.53 (1.27, 1.84)	<0.05
		Anticipated (healthcare provider)		1.09 (0.86, 1.38)	>0.05
		Enacted		1.90 (1.35, 2.67)	<0.05
		Anticipated^a^		1.98 (1.33, 2.96)	<0.05
		Anticipated^b^		1.45 (1.03, 2.05)	<0.05
		Internalised		2.67 (1.86, 3.75)	<0.05
Felker-Kantor et al. (2019), United States	Cross-sectional	Perceived	HADS; higher score on depression scale	2.05 (1.25, 3.35)^c^	<0.01
		Personalised/enacted		1.72 (0.99, 2.90)^c^	>0.05
		Anticipated^a^		1.44 (0.91, 2.27)^c^	>0.05
		Anticipated^b^		1.70 (1.09, 2.66)^c^	<0.05
		Internalised		1.93 (1.29, 2.90)^c^	<0.001
Li et al. (2016), China	Cross-sectional	Enacted	CES-D; higher score on depression scale	8.59 (2.55, 28.89)	0.001
Tao et al. (2017), China	Cross-sectional	Perceived	HADS; classified as having borderline or suspected depression	1.05 (1.03, 1.07)^d^	NR
		Vicarious		1.08 (1.05, 1.12)^d^	
		Vicarious (healthcare)		1.13 (1.07, 1.20)^d^	
		Vicarious (community/family)		1.19 (1.12, 1.28)^d^	
		Internalised		1.11 (1.08, 1.14)^d^	
		Internalised (contact avoidance)		1.18 (1.12, 1.23)^d^	
		Internalised (shame/guilt)		1.21 (1.15, 1.26)^d^	
		Unspecified		1.04 (1.03, 1.05)^d^	
Liu et al. (2018), China	Cross-sectional	Perceived	Burns Depression Checklist; higher score on depression scale (score >11)	1.11 (1.06, 1.16)	NR
**Adjusted analysis**					
Drumright et al. (2023), United States	Longitudinal	Internalised	NR; higher score on depression scale	1.60 (1.39, 1.85)^e^	NR
				1.36 (1.27, 1.45)^f^	
**Unstandardised analysis**				**Linear (high vs low stigma levels),** ***β* value (95% CI)**	
Kay et al. (2018), United States	Cross-sectional	Enacted (community)	PHQ-9; higher score on depression scale	1.75 (0.50)^g^	<0.001
		Enacted (healthcare)		1.76 (0.79)^g^	0.03
Molina et al. (2018), United States	Cross-sectional	Enacted	PHQ-8; higher score on depression scale	0.45 (NR)	<0.001
Turan et al. (2022), United States	Cross-sectional	Enacted (healthcare)	CES-D; higher score on depression scale	3.78 (2.50, 5.07)^h^	NR
				3.10 (1.77, 4.43)^i^	
		Anticipated (healthcare)		1.5 (0.83, 2.16)^h^	
				1.31 (0.64, 1.98)^i^	
Rendina et al. (2018), United States	Cross-sectional	Anticipated (situational)	ODSIS; experiencing depression concurrently to stigma event	0.01 (−0.06, 0.08)	>0.05
		Anticipated (individual)		0.03 (-0.20, 0.26)	>0.05
		Anticipated (situational)	Subsequent nighttime experiences of depression after afternoon stigma event	0.04 (−0.03, 0.10)	>0.05
		Anticipated (individual)		−0.02 (−0.25, 0.21)	>0.05
		Internalised (situational)	Experiencing depression concurrently to stigma event	0.22 (0.11, 0.33)	<0.01
		Internalised (individual)		0.24 (-0.01, 0.48)	>0.05
		Internalised (situational)	Subsequent nighttime experiences of depression after afternoon stigma event	0.11 (0.01, 0.22)	<0.05
		Internalised (individual)		0.31 (0.00, 0.62)	<0.05
Brown et al. (2016), United States	Cross-sectional	Personalised	CCAD; more depression symptoms	0.92 (0.65, 1.19)	<0.05
		Anticipated^a^		1.26 (0.75, 1.76)	<0.05
		Internalised		1.63 (1.31, 1.96)	<0.05
		Anticipated^b^		0.91 (0.66, 1.17)	<0.05
		Unspecified		0.52 (0.39, 0.65)	<0.05
		Personalised	CES-D; more depression symptoms	0.52 (0.35, 0.69)	<0.05
		Anticipated^a^		0.71 (0.39, 1.02)	<0.05
		Internalised		0.92 (0.71, 0.13)	<0.05
		Anticipated^b^		0.52 (0.36, 0.68)	<0.05
		Unspecified		0.29 (0.21, 0.38)	<0.05
Hong et al. (2023), United States	Cross-sectional	Unspecified	PHQ-9; higher score on depression scale	0.24 (0.05)^g^	<0.001
Hussen et al. (2022), United States	Cross-sectional	Unspecified	CESD-R; higher score on depression scale	10.31 (NR)	0.012
Murphy et al. (2018), Europe	Cross-sectional	Enacted	HADS; higher score on depression scale	0.15 (0.02, 0.28)	NR
		Anticipated		−0.08 (−0.26, 0.09)	
		Internalised		0.4 (0.26, 0.54)	
Yang et al. (2020), China	Cross-sectional	Internalised	CESD-10; higher score on depression scale	4.40 (0.74)^g^	<0.001
Liu et al. (2018), China	Cross-sectional	Perceived	Burns Depression Checklist; higher score on depression scale (score >11)	0.10 (0.02)^g^	<0.001
**Standardised analysis**					
Walsh et al. (2023), United States	Cross-sectional	Perceived	PHQ-9; higher score on depression scale	0.29 (0.05)^j^	<0.001
				0.12 (0.05)^k^	0.003
Mitzel et al. (2015), United States	Cross-sectional	Unspecified	CES-D; higher score on depression scale	0.49 (NR)	<0.01
Hussen et al. (2022), United States	Cross-sectional	Unspecified	CESD-R; higher score on depression scale	5.43 (2.12)^g^	0.012
Murphy et al. (2018), Europe	Cross-sectional	Enacted	HADS; higher score on depression scale	0.15 (NR)	<0.05
		Anticipated^b^		−0.06 (NR)	NR
		Internalised		0.36 (NR)	<0.001
Zhang et al. (2016), China	Cross-sectional	Perceived	CESD-10; higher score on depression scale	0.20 (0.15, 0.25)	<0.0001
		Internalised		0.29 (0.25, 0.32)	<0.0001
		Enacted		2.23 (1.86, 2.61)	<0.0001

CCAD, Costello-Comrey Anxiety and Depression scale; CES-D, Center for Epidemiologic Studies Depression Scale; CESD-R, Center for Epidemiologic Studies Depression Scale-Revised; CESD-10, Center for Epidemiologic Studies Depression Scale, 10- item version; HADS, Hospital Anxiety and Depression Scale; IHS, internalized HIV stigma; NR, not reported; ODSIS, Overall Depression Severity and Impairment Scale; OR, odds ratio; PHQ-8, Patient Health Questionnaire-8; PHQ-9, Patient Health Questionnaire-9.

a Anticipated stigma reported as “disclosure concerns.” ^b^Anticipated stigma reported as “concern with public attitudes.” ^c^Relative risk ratio (95% CI). ^d^One-point increase in stigma score versus one-point increase in the HADS scale. ^e^Adjusted rate ratio: conditional logistic regression for IHS score >2. ^f^Adjusted rate ratio: Poisson regression stigma score. ^g^Standard error (SE). ^h^Regression coefficients reported from moderation analysis examining the association between experienced stigma and resilience, and the effect their interaction has on depressive symptoms. ^i^Regression coefficients reported from moderation analysis examining the association between experienced stigma and optimism, and the effect their interaction has on depressive symptoms. ^j^Simple linear regression, explored bivariate associations; standard error in parentheses. ^k^Multiple linear regression; considered predictors simultaneously; standard error in parentheses.

All 9 studies that assessed higher internalised stigma levels and depression found significant associations (United States, *n* = 5 (Brown et al., 2016; Chapman Lambert et al., 2020; Drumright et al., 2023; Felker-Kantor et al., 2019; Rendina et al., 2018); Europe, *n* = 1 (Murphy et al., 2018); China, *n* = 3 (Tao et al., 2017; Yang et al., 2020; Zhang et al., 2016)), with 5 showing consistent findings in specific populations of people with HIV, including women, (Brown et al., 2016) men who have sex with men (Murphy et al., 2018; Tao et al., 2017; Yang et al., 2020), and gay and bisexual men (Rendina et al., 2018). Additionally, significant associations with higher unspecified or overall HIV-related stigma levels were found in several populations of people with HIV, including women (Brown et al., 2016), gay, bisexual, and other Black men who have sex with men (Hong et al., 2023; Hussen et al., 2022), and men who have sex with men (Mitzel et al., 2015; Tao et al., 2017).

Internalised HIV-related stigma was also found to be associated with increased anxiety in all assessed studies (United States, *n* = 5 (Blake Helms et al., 2017; Brown et al., 2016; Drumright et al., 2023; Felker-Kantor et al., 2019; Rendina et al., 2018); Europe, *n* = 1 (Murphy et al., 2018); China, *n* = 2 (Yang et al., 2020; Zhang et al., 2016)). Furthermore, unspecified stigma was associated with severe but not moderate anxiety in specific populations of people with HIV, including young Black men who have sex with men as well as young, Black gay and bisexual men (Hussen et al., 2022).

### HIV-related stigma and QoL

Associations between HIV-related stigma and HRQoL or QoL outcomes were identified in 27 studies in the United States (*n* = 12) (Brown et al., 2023; Chapman Lambert et al., 2020; Cramer et al., 2017; Fekete et al., 2016; Hong et al., 2023; Hussen et al., 2022; Kamen et al., 2016; Kay et al., 2018; Slater et al., 2013; Slater et al., 2015; Travaglini et al., 2018; Walsh et al., 2023), Europe (*n* = 6) (de Daas et al., 2019; Dibb, 2018; Drewes et al., 2021; Fumaz et al., 2019; Nilsson Schönnesson et al., 2022; Stutterheim et al., 2022), China (*n* = 8) (Chen et al., 2022; Liu et al., 2013; Song et al., 2016; Wen et al., 2013; Yang et al., 2020; Zhang et al., 2016; Zhao et al., 2019; Zhu et al., 2020), and Brazil (*n* = 1) (Kerrigan et al., 2017). Among the 27 studies, 19 different HRQoL measurement tools were used to assess general (16) and HIV-related (3) HRQoL. HIV-related stigma was measured using validated tools, excluding 2 studies where reporting was unclear (Fumaz et al., 2019; Liu et al., 2013), and 1 study measured stigma using a tool that was not specific to the context of HIV-related stigma (Kay et al., 2018).

Internalised (*n* = 7) (Chapman Lambert et al., 2020; Chen et al., 2022; Cramer et al., 2017; Dibb, 2018; Drewes et al., 2021; Nilsson Schönnesson et al., 2022; Zhang et al., 2016) and perceived (*n* = 7) (Chen et al., 2022; Dibb, 2018; Kamen et al., 2016; Liu et al., 2013; Stutterheim et al., 2022; Wen et al., 2013; Zhang et al., 2016) HIV-related stigma were the most reported stigma types associated with general HRQoL. Higher internalised HIV-related stigma levels were significantly associated with lower general HRQoL (*n* = 5) (Chapman Lambert et al., 2020; Chen et al., 2022; Cramer et al., 2017; Drewes et al., 2021; Zhang et al., 2016) and lower HIV-related QoL (*n* = 6) (Brown et al., 2023; Fumaz et al., 2019; Slater et al., 2013; Slater et al., 2015; Yang et al., 2020; Zhu et al., 2020). Additionally, higher perceived stigma levels were significantly associated with lower general HRQoL (*n* = 6) (Chen et al., 2022; Kamen et al., 2016; Liu et al., 2013; Stutterheim et al., 2022; Wen et al., 2013; Zhang et al., 2016) and lower HIV-related HRQoL (*n* = 2) (Song et al., 2016; Walsh et al., 2023). Of 6 studies, 5 revealed significant associations between higher HIV-related stigma levels and worsened social or family functioning (Greene et al., 2013; Kay et al., 2018; Nilsson Schönnesson et al., 2022; Wang et al., 2019; Wen et al., 2013). This SLR did not identify any studies reporting on the association between HIV-related stigma and discrimination, and only 1 study suggested that higher HIV-related enacted stigma levels are associated with increased unemployment, increased part-time work, and lower likelihood of making a median to high income (Zhang et al., 2016).

### Economic outcomes and medication use for comorbid conditions

No studies were identified by this SLR that reported an association between HIV-related stigma and interventions for comorbid conditions or healthcare resource utilisation and direct or indirect costs (economic outcomes).

### Drug, substance, and alcohol use

Six studies reported data on the association between HIV-related stigma and drug, substance, and alcohol use (United States, *n* = 5 (Drumright et al., 2023; Felker-Kantor et al., 2019; Lipira et al., 2020; Schafer et al., 2022; Sizemore et al., 2020); China, *n* = 1 (Zhang et al., 2016)). All studies evaluated for drug, substance, and alcohol use employed validated scales to measure HIV stigma.

Data were limited and inconsistent, which may be due to differing methodologies, study populations, and countries of origin. For example, in US studies, higher internalised HIV-related stigma levels were associated with binge alcohol consumption (adjusted rate ratio [95% CI], 1.32 [1.12, 1.55]), at-risk alcohol consumption (1.15 [1.01, 1.31]) (Drumright et al., 2023), and significantly increased risk of hazardous drinking (relative risk [95% CI], 1.60 [1.17, 2.19]; *P* < 0.01) (Felker-Kantor et al., 2019). Conversely, the study conducted in China reported that higher internalised HIV-related stigma levels marginally decreased the chance of using alcohol; although significance should be interpreted with caution as the 95% CI of the adjusted OR touches the line of null effect (adjusted OR [95% CI], 0.98 [0.96, 1.0]; *P* < 0.05) (Zhang et al., 2016). Statistically significant associations with alcohol use and enacted, anticipated, perceived, or unspecified HIV-related stigma were not found.

A US study found that higher internalised HIV-related stigma levels were associated with use of methamphetamine (adjusted rate ratio [95% CI], 1.35 [1.12, 1.62]) and opioids (1.50 [1.04, 2.16]), but not cocaine (1.13 [0.89, 1.44]) (Drumright et al., 2023). Higher unspecified HIV-related stigma levels were significantly associated with recreational marijuana use in the past 30 days in gay men, bisexual men, men who have sex with men, and transgender women newly diagnosed with HIV in another US study (OR [95% CI], 1.07 [1.02, 1.12]; *P* = 0.007) (Schafer et al., 2022). Additionally, high unspecified stigma levels were significantly associated with substance use in adults aged ≥50 years with HIV (standardised *β*, 0.22; *P* = 0.029) (Sizemore et al., 2020). No significant associations between high HIV-related stigma (internalised, enacted, or perceived) levels and drug use were observed in the study conducted in China (Zhang et al., 2016).

## Discussion

HIV-related stigma remains an ongoing international challenge. Despite global efforts to reduce stigma and discrimination among people with HIV, we found substantial evidence to support that HIV-related stigma remains prevalent and is negatively associated with clinical and QoL outcomes among people with HIV. Among identified studies, HIV-related stigma was associated with reduced HIV-specific healthcare engagement and retention in 73% (8/11), ART adherence in 75% (15/20), virologic suppression in 79% (11/14), and HIV-related QoL in 86% (6/7) of studies. Furthermore, HIV-related stigma was significantly associated with depression (15/15) and anxiety (10/10) in all studies reporting *P* values. The data identified in this SLR suggest that the development and implementation of effective interventions to address HIV-related stigma remain an unmet need.

Internalised HIV-related stigma was the most frequently assessed form of HIV-related stigma identified across the 4 research questions studied. Hedge et al noted that internalised stigma continues to be an issue among people with HIV, while other types of stigma, such as enacted stigma, may be less frequent or severe than experienced in the past (Hedge et al., 2021). Internalised stigma was also associated with compromised healthcare, a reluctance to engage with HIV testing services, and disengagement from medical services among people with HIV and disengagement from HIV prevention services among individuals with a greater likelihood of HIV acquisition. High HIV-related internalised stigma prevalence (72%) was identified in a study from China (Zhang et al., 2015), with ranges of internalised stigma prevalence in the United States and Europe of 11% to 35% (Drumright et al., 2023; Kota et al., 2023; Pearson et al., 2021; Williams et al., 2022) and 27% to 57%, respectively (Noori et al., 2022). These data suggest there may be cultural differences between Eastern and Western populations that may affect the experience of internalised HIV-related stigma and highlight the importance of considering cultural factors when measuring and interpreting prevalence across geographic regions.

Distinct cultural frameworks likely drive these geographic disparities (Aung et al., 2023; Handayani et al., 2021). In Eastern cultures, religious beliefs (eg, karma, divine punishment) cause individuals to internalise an HIV diagnosis as a moral failing (Aung et al., 2023; Handayani et al., 2021; Raya & Nilmanat, 2021). Furthermore, collectivist norms and the preservation of social standing inextricably link personal standing to family reputation (Handayani et al., 2021). This dynamic transforms an HIV diagnosis into a familial threat, leading to what is known as a ‘spoiled identity’ within the community (Raya & Nilmanat, 2021), and driving more severe self-stigmatisation than in Western societies, where individualistic values and HIV advocacy offer a buffer (Operario et al., 2022). However, as the prevalence data from China identified in this SLR date back to 2015, comparisons with more recent Western data should be interpreted with caution. The overall prevalence of HIV-related stigma was broad across stigma types and geographic regions; yet the methods used to measure prevalence were inconsistent across included studies, making it difficult to draw accurate comparisons. A plethora of measures to assess HIV-related stigma exist, with a recent SLR reporting 19 different PROMs utilised across 45 studies (Zhang et al., 2024). Due to the many methods used to assess HIV-related stigma, comparability across studies is challenging; thus, having a common starting point could promote internationally comparable data on HIV-related stigma (Ferguson et al., 2022). Importantly, accuracy and reproducibility are key to obtaining reliable and valid results when selecting PROMs (Zhang et al., 2024). However, researchers and clinicians are limited when selecting the most appropriate PROM by the lack of data on the different types of HIV-related stigma tools utilised in people with HIV across geographies and populations.

These data emphasise the importance of employing standardised, validated measures to accurately track and compare the prevalence of HIV-related stigma across geographic regions and populations in future studies. Additionally, high HIV-related stigma levels were reported by specific populations including transgender women and men who have sex with men, which may be attributed to intersecting stigmas related to gender identity and sexual orientation (Layland et al., 2020).

Among UNAIDS’ top-line targets for 2025 is for 95% of people with HIV to be on treatment and 95% to maintain viral suppression (UNAIDS, 2020). Our findings support the hypothesis that HIV-related stigma hinders achievement of these targets. Higher HIV-related stigma levels across the various forms were associated with reduced HIV care engagement and retention, suboptimal treatment adherence, and unsuppressed viral load. Engagement with the HIV care continuum can be an overwhelming experience for people with HIV and involves initiating lifelong ART, regular HIV clinic visits, and shared decision-making about managing their HIV (Chapman Lambert et al., 2020).

Switching from daily oral ART to a cabotegravir plus rilpivirine long-acting (CAB+RPV LA) regimen has been shown to reduce the proportion of participants feeling stigmatised by HIV treatment (Valenti et al., 2024). At baseline, 18% of participants switching from daily oral ART to CAB+RPV LA reported feeling always or often stigmatised by HIV treatment (*N* = 308), and by Month 12 of CAB+RPV LA treatment, this number was reduced to <2% of participants (*N* = 229). Qualitative literature suggests this reduction is primarily driven by the elimination of daily pills, which significantly alleviates concerns regarding unintentional status disclosure (Erguera et al., 2024; Kerrigan et al., 2018). Removing these medications relieves the daily psychological burden and constant mental reminders of living with a stigmatised illness, allowing individuals to create psychological distance from the daily experience of living with HIV (Erguera et al., 2024). Furthermore, participants receiving CAB+RPV LA had improved adherence (74%; *N* = 1245) compared with participants receiving oral ART (30%; *N* = 1275), evaluated by proportion of days covered (Garris et al., 2024). These data suggest that switching from daily oral ART to a long-acting injectable may be a treatment option to increase adherence and engagement in care by reducing HIV-related stigma.

Enacted HIV-related stigma in the healthcare setting can manifest as refusal of treatment and failure to protect HIV status confidentiality, whereas enacted stigma in community settings may manifest as expression of negative attitudes toward people with HIV and social isolation and rejection in places of public accommodation (Kay et al., 2018). Importantly, this SLR identified associations between high enacted stigma levels in the healthcare setting and unsuppressed viral load, which have important implications for healthcare providers to recognise and mitigate HIV-related stigma in healthcare settings.

Consistent with previous research on HIV-related stigma and prevalence of mental health conditions (Chambers et al., 2015; Rueda et al., 2016), our findings indicate that high HIV-related stigma levels are associated with an increased prevalence of mental health conditions, including depression, anxiety, suicidal ideation, and post-traumatic stress disorder. Additionally, mental health conditions, including major depression, among people with HIV have been associated with increased morbidity, mortality, and worsened outcomes along the entire HIV care continuum (Fuenmayor & Cournos, 2022). This SLR did not identify any evidence linking HIV-related stigma with physical conditions of interest, including cardiovascular disease, hypertension, diabetes, dyslipidemia, obesity, chronic renal disease, metabolic syndrome, or bone disease, indicating an area of future research focus.

Reducing HIV-related stigma and improving HRQoL among people with HIV are key to achieving the UNAIDS well-being targets (UNAIDS, 2020). In studies examining the association between HIV-related stigma and HRQoL, data trends indicate that higher stigma levels are associated with worsened general and HIV-specific HRQoL outcomes. Previous research has demonstrated that HIV is associated with lower HRQoL (Bing et al., 2000; Liu et al., 2006), and HRQoL is directly associated with clinical outcomes in people with HIV (Call et al., 2000; Penedo et al., 2003). The presence of social support can greatly impact the QoL in people with HIV by contributing to the development of resilience and combating the mental health aspects of the disease; nevertheless, HIV-related stigma within social networks of people with HIV can result in strained relationships and isolation (Brown et al., 2023). Our data support this result, as this SLR identified trends between higher HIV-related stigma levels across various forms and worsened social functioning outcomes.

HIV-related stigma profoundly impairs social cognition and social functioning (Akınkoç et al., 2025; Earnshaw & Chaudoir, 2009; Turan et al., 2017). Anticipating stigma triggers defensive cognitive adaptations, including hypervigilance to rejection and pervasive mistrust (Turan et al., 2017). This altered cognition drives behavioural withdrawal, self-isolation, and the breakdown of support networks (Rueda et al., 2016; Turan et al., 2017). As our findings indicate, this isolation extends into broader societal participation, creating significant barriers to employment, career advancement, and engagement with the healthcare system (Aung et al., 2023; Rueda et al., 2016). Ultimately, this degradation of social functioning acts as a primary catalyst for the worsened mental health and clinical outcomes observed across the literature (Rueda et al., 2016; Turan et al., 2017).

Drug, substance, and alcohol use are more common in people with HIV compared with the general population (Galvan et al., 2002; Justice et al., 2010; Shiau et al., 2017), which is of particular interest as they have been associated with HIV-related mortality (High et al., 2012), suboptimal antiretroviral adherence (Hendershot et al., 2009), and increased risk of HIV transmission (Scott-Sheldon et al., 2013; Xu et al., 2014). However, the evidence identified in this SLR was limited and inconsistent. Some studies reporting a significant association focused on specific populations such as gay men, bisexual men, men who have sex with men, and transgender women who may be more prone to substance use due to intersectional stigma (Earnshaw, 2020). In contrast, a study from China reported that higher internalised and perceived HIV-related stigma levels marginally decreased the likelihood of alcohol use (Zhang et al., 2016). The authors suggested that social alcohol consumption is common in China and people with HIV may employ coping strategies other than alcohol use, as people with HIV on ART are advised to moderate their alcohol intake to avoid medication side effects. Although this SLR only identified 1 study reporting the association of HIV-related stigma and employment, Perri et al found that HIV-related stigma was described as the greatest barrier to gaining employment among 35 interviews of people with HIV (Perri et al., 2021). Additionally, stigma or discrimination due to HIV status hinders access to employment, job retention, and career advancement, according to a report by the International Labour Organisation (Internal Labour Organisation, 2018). Thus, HIV-related stigma or discrimination can lead to job loss and prevent career progression in people with HIV.

The findings of this SLR emphasise the extensive impact that HIV-related stigma has on the lives of people with HIV and demonstrate that the development and implementation of effective tools to tackle stigma are imperative to achieving the UNAIDS 2030 targets. Discussions regarding stigma should be implemented in the healthcare setting and regularly incorporated into routine clinic visits with people with HIV, as there is increasing evidence on the value of supportive and de-stigmatising HIV services in differing sociocultural settings (Nyblade et al., 2009). Importantly, a specific instrument or PROM may not be necessary to alleviate HIV-related stigma in the healthcare setting, as these measures are generally used to provide evidence of HIV-related stigma at the population level (Antela et al., 2022). Opening lines of communication regarding stigma in patient-provider conversations may be an effective tool to attenuate HIV-related stigma and promote the achievement of the UNAIDS 95-95-95 targets. At the individual level, increasing awareness among healthcare workers of what HIV-related stigma is and the benefits of reducing it may open lines of communication between healthcare workers and people with HIV (Nyblade et al., 2009). Additionally, stigma interventions in the healthcare setting should address fears around occupational acquisition and provide opportunities for healthcare workers to reflect on attitudes toward people with HIV (Geter et al., 2018; Stutterheim et al., 2014). Interventions focused on building knowledge around HIV generally reduced HIV-related stigma (Loutfy et al., 2015); therefore, people with HIV who have better coping strategies, education, and more support may experience reduced HIV-related stigma. Specifically, individual-level interventions like resilience training utilise adaptive cognitive-behavioural skills to mitigate the psychological impact of both internalised and enacted stigma (Li et al., 2022).

While the majority of studies used validated tools, such as the Berger HIV stigma scale, a number of studies utilised non-validated, author-developed scales, thus rendering comparisons across studies difficult as reliability in data may differ. Researchers should be encouraged to use newer, more contemporary validated tools to support consistent reporting and ensure comparisons in data are feasible across studies.

Although this SLR utilised robust methodology and provided a large overview of several topics connected to HIV-related stigma, it does have limitations. It is possible that some publications may not have been identified and more recent evidence may have been published after May 2023. Due to the large number of studies identified, the evidence was refined so that only studies from the United States, Europe, China, and Brazil were included in data synthesis. These specific regions were purposefully selected to capture a diverse cross-section of socioeconomic statuses, cultural frameworks, and healthcare system contexts. Therefore, only a portion of global evidence has been examined. The geographic scope was limited to the United States and Europe (high-income), and Brazil and China (upper-middle income). Although these regions provide a diverse outlook of global healthcare infrastructures, the exclusion of low- and middle-income countries remains a limitation and may affect global generalisability of findings. Despite these limitations, this review captured a broad evidence base and provides a starting point for further research into individual topics of interest pertaining to HIV-related stigma.

In conclusion, the substantial evidence identified by this SLR suggests that HIV-related stigma is a significant issue for people with HIV and negatively impacts QoL, mental health, employment, and clinical outcomes. These findings also underscore the importance of integrating stigma considerations into the dialogue between physicians and people with HIV to foster shared decision-making on treatment and support. We identified several evidence gaps, including associations between HIV-related stigma and the prevalence of physical comorbid conditions and interventions for these conditions, discrimination, and economic outcomes.

Considerations for future research include the development of holistic interventions that combine individually targeted strategies (such as resilience training) with broader structural interventions. These considerations allow for exploration of intersectionality, as HIV-related stigma is frequently compounded by intersecting stigmas related to race, gender identity, sexual orientation, and socioeconomic status. Furthermore, while individual coping mechanisms can mitigate personal burden, the broader structural context remains a key driver of discrimination. Addressing stigma at the institutional and policy level is likely necessary to achieve a meaningful reduction in prevalence. Future work could further account for governance diversity across geographic settings, recognising how varying state laws, healthcare policies, and political frameworks either mitigate or reinforce societal stigma against people with HIV.

## Supplementary Material

Supplemental Table 3.docx Supplemental Table 3.docx

Supplemental Table 2.docx Supplemental Table 2.docx

Supplemental Table 1.docx Supplemental Table 1.docx

Supplemental Table 4.docx Supplemental Table 4.docx

## Data Availability

Anonymized individual participant data and study documents can be requested for further research from www.clinicalstudydatarequest.com.

## Prior publication

Data included in this manuscript have previously been presented in part at Continuum 2024; June 9-11, 2024; Fajardo, Puerto Rico; Poster 1036.
