# Supplementary material for: The burden of HIV-related stigma on clinical and quality of life outcomes: results from a systematic literature review
Source: Health Psychol Behav Med. 2026 Jul 28;14(1):2672790. doi: 10.1080/21642850.2026.2672790 (PMC13421116; doi:10.1080/21642850.2026.2672790)
Supplement: Supplemental Table 3.docx [file RHPB_A_2672790_SM2257.docx]

| **Supplemental Table 3.** OVID MEDLINE^®^ and Embase^®^ Search Strategies for Identifying Evidence for the Association Between HIV-Related Stigma and the Humanistic Burden, Clinical Outcomes, and Treatment Concepts and Mediators for Concepts Associated With HIV-Related Stigma (Date of Search: May 25, 2023) | | |
| --- | --- | --- |
| **Search strategy number** | **Search terms (MEDLINE^®^**) | **Number of records** |
| **Population experiencing HIV-related stigma** | | |
| 1 | HIV/ or HIV Infections/ | 233,921 |
| 2 | ((human immunodeficiency adj2 virus$) or (human immun? deficiency adj2 virus$) or acquired immun? deficiency syndrome virus$ or acquired immunodeficiency syndrome virus$ or (aids associated adj (lentivirus$ or retrovirus$ or virus$)) or aids related virus$ or aids virus$ or HIV$ or human t cell lymphotropic virus type iii or immunodeficiency associated virus$ or lav or PLHIV or ALHIV or PLWHA or (lymphadenopathy associated adj2 (retrovirus$ or virus$))).ti,ab,kf. /freq=3 | 221,905 |
| 3 | or/1-2 | 311,823 |
| 4 | Social Stigma/ or Prejudice/ or Perception/ or Taboo/ or Attitude/ or Social Isolation/ | 146,677 |
| 5 | (Stigma$ or ostraci$ or selfdiscriminat$ or discriminat$ or "fear of outing" or "fear of coming out" or ((perceived or actual or fear or felt or anticipated) adj7 (judg$ or prejudice$)) or "not accept$" or non accept$ or nonaccept$ or unaccept$ or un accept$ or victimi?ation or psychosocial factor$ or psycho-social factor$ or social alienat$ or marginal$).ti,ab,kf. | 515,989 |
| 6 | or/4-5 | 639,528 |
| 7 | 3 and 6 | 17,213 |
| **Outcomes** | | |
| 8 | "Quality of Life"/ or Quality-Adjusted Life Years/ | 278,692 |
| 9 | (QALY or QALYs or HRQoL$ or quality-adjusted-life-year$ or "quality of life" or utility or utilities).ti,ab,kf. | 623,934 |
| 10 | Mental Health/ | 60,536 |
| 11 | (Mental adj2 (condition$ or health or factor$ or state$ or status)).ti,ab,kf. | 267,566 |
| 12 | Treatment Outcome/ or Outcome Assessment, Health Care/ | 1,221,787 |
| 13 | (((clinical or treatment$ or therap$ or patient$) adj2 (outcome$ or assessment$)) or "outcome and process assessment$" or outcome management$ or (outcome$ adj2 assessment$)).ti,ab,kf. | 683,444 |
| 14 | Comorbidity/ or Multimorbidity/ | 126,833 |
| 15 | (comorbid$ or co-morbid$ or multi-morbid$ or multimorbid$ or multidisease$ or multi-disease$ or ((multiple or co-existing) adj (disease$ or illness$ or condition$ or disorder$))).ti,ab,kf. | 270,256 |
| 16 | Cardiovascular Diseases/ | 175,703 |
| 17 | (((cardiovascular or cardio-vascular angiocardiovascular) adj2 (disease$ or complication$ or disorder$ or disturbance$ or lesion$ or syndrome$ or event$)) or angiocardiopath$).ti,ab,kf. | 289,853 |
| 18 | Myocardial Infarction/ | 178,727 |
| 19 | ((heart or cardiac or cardial or myocardial or myocardium) adj2 (infarct$ or attack$)).ti,ab,kf. | 235,918 |
| 20 | Stroke/ | 131,004 |
| 21 | (((cerebrovascular or cerebro-vascular or brain or cerebral or cerebrum) adj2 (accident$ or lesion$ or attack$ or insult$ or insufficienc$ or arrest$ or fail$ or injur$)) or stroke or apoplex$).ti,ab,kf. | 424,727 |
| 22 | Heart Failure/ | 142,774 |
| 23 | ((heart or cardiac or cardial or myocardial) adj2 (fail$ or decompensation or incompeten$ or insuffien$ or stand still)).ti,ab,kf. | 231,459 |
| 24 | Hypertension/ | 256,913 |
| 25 | ((hypertensive adj1 (disease$ or effect$ or response$)) or hypertension or high blood pressure).ti,ab,kf. | 469,592 |
| 26 | Kidney Failure, Chronic/ | 100,721 |
| 27 | ((Chronic adj (kidney or renal) adj (failure$ or disease$ or insufficienc$)) or chronic nephropath$ or ckd or esrd).ti,ab,kf. | 124,721 |
| 28 | Metabolic Syndrome/ | 38,046 |
| 29 | ((insulin resistance or metabolic) adj2 resistance).ti,ab,kf. | 99,170 |
| 30 | Dyslipidemias/ | 14,233 |
| 31 | (dyslip?emia$ or dyslipid?emia$ or dys-lip?emia$ or dys-lipid?emia$).ti,ab,kf. | 44,568 |
| 32 | Diabetes Mellitus, Type 2/ | 169,491 |
| 33 | (T2dm or TiiDM or Tii-DM or "type 2 DM" or type-ii-DM or DM2 or DM-2 or "T2-DM" or NIDDM or (diabet$ adj3 ("typ$ 2" or "typ$ ii" or type2 or typeii or "typ$ two"))).ti,ab,kf. | 198,754 |
| 34 | (noninsulin$ depend$ or non-insulin$ depend).ti,ab,kf. | 1479 |
| 35 | Obesity/ | 214,585 |
| 36 | (obese or obesity or adipose tissue hyperplasia or adipositas or adiposity or excess body weight or corpulen$ or fat overload syndrome$ or obesitas or overweight or over-weight).ti,ab,kf. | 412,500 |
| 37 | Bone Diseases/ | 23,062 |
| 38 | ((Bone or skeletal or skeleton) adj (disease$ or disorder$)).ti,ab,kf. | 26,075 |
| 39 | Biomarkers/ | 346,965 |
| 40 | (Biological marker$ or biomarker$ or bio marker$).ti,ab,kf. | 416,284 |
| 41 | Health services accessibility/ | 85,232 |
| 42 | (access$ adj2 (medication$ or healthcare or care or health service$ or medicine$ or therap$ or treatment$ or program$)).ti,ab,kf. | 57,354 |
| 43 | "patient acceptance of health care"/ or patient compliance/ or medication adherence/ | 136,721 |
| 44 | ((therap$ or treatment$ or medication$ or medicine$ or patient$ or client$ or drug$) adj2 (adher$ or compl$ or noncompl$ or nonadher$ or persisten$ or participat$ or collaborat$)).ti,ab,kf. | 433,990 |
| 45 | Patient satisfaction/ or patient acceptance of health care/ | 141,845 |
| 46 | ((accept$ or nonaccept$) adj2 (health care or healthcare)).ti,ab,kw. | 753 |
| 47 | (((patient$ or treatment$) adj2 satisf$) or desire$ or view$).ti,ab,kw. | 761,877 |
| 48 | or/8-47 | 6,150,374 |
| 49 | (link$ or associat$ or mediat$ or predict$ or determinant$ or correlat$).ti,ab,kf. /freq=3 | 2,889,080 |
| 50 | 7 and 48 and 49 | 1717 |
| 51 | (letter or comment or editorial).pt. | 2,160,281 |
| 52 | 50 not 51 | 1714 |
| **Search strategy number** | **Search terms (Embase^®^)** | **Number of records** |
| **Population experiencing HIV-related stigma** | | |
| 1 | *Human immunodeficiency virus/ or *Human immunodeficiency virus infection/ or *Human immunodeficiency virus infected patient/ | 264,312 |
| 2 | ((human immunodeficiency adj2 virus$) or (human immun? deficiency adj2 virus$) or acquired immun? deficiency syndrome virus$ or acquired immunodeficiency syndrome virus$ or (aids associated adj (lentivirus$ or retrovirus$ or virus$)) or aids related virus$ or aids virus$ or HIV$ or human t cell lymphotropic virus type iii or immunodeficiency associated virus$ or lav or PLHIV or ALHIV or PLWHA or (lymphadenopathy associated adj2 (retrovirus$ or virus$))).ti,ab,kw. /freq=3 | 276,910 |
| 3 | or/1-2 | 373,399 |
| 4 | Stigma/ or social stigma/ or perception/ or psychological well-being/ or taboo/ or social attitude/ or social isolation/ or social exclusion/ | 257,980 |
| 5 | (Stigma$ or ostraci$ or selfdiscriminat$ or discriminat$ or "fear of outing" or "fear of coming out" or ((perceived or actual or fear or felt or anticipated) adj7 (judg$ or prejudice$)) or "not accept$" or non accept$ or nonaccept$ or unaccept$ or un accept$ or victimi?ation or psychosocial factor$ or psycho-social factor$ or social alienat$ or marginal$).ti,ab,kw. | 656,484 |
| 6 | or/4-5 | 875,084 |
| 7 | 3 and 6 | 20,100 |
| **Outcomes** | | |
| 8 | Quality of life/ or quality adjusted life year/ | 646,541 |
| 9 | (QALY or QALYs or HRQoL$ or quality-adjusted-life-year$ or "quality of life" or utility or utilities).ti,ab,kw. | 952,951 |
| 10 | mental health/ | 203,467 |
| 11 | (Mental adj2 (condition$ or health or factor$ or state$ or status)).ti,ab,kw. | 329,441 |
| 12 | Clinical outcome/ or treatment outcome/ or outcome assessment/ | 1,982,551 |
| 13 | (((clinical or treatment$ or therap$ or patient$) adj2 (outcome$ or assessment$)) or "outcome and process assessment$" or outcome management$ or (outcome$ adj2 assessment$)).ti,ab,kw. | 1,097,892 |
| 14 | Comorbidity/ | 385,402 |
| 15 | (comorbid$ or co-morbid$ or multi-morbid$ or multimorbid$ or multidisease$ or multi-disease$ or ((multiple or co-existing) adj (disease$ or illness$ or condition$ or disorder$))).ti,ab,kw. | 486,621 |
| 16 | cardiovascular disease/ | 341,115 |
| 17 | (((cardiovascular or cardio-vascular angiocardiovascular) adj2 (disease$ or complication$ or disorder$ or disturbance$ or lesion$ or syndrome$ or event$)) or angiocardiopath$).ti,ab,kw. | 421,459 |
| 18 | heart infarction/ | 308,601 |
| 19 | ((heart or cardiac or cardial or myocardial or myocardium) adj2 (infarct$ or attack$)).ti,ab,kw. | 336,798 |
| 20 | cerebrovascular accident/ | 283,902 |
| 21 | (((cerebrovascular or cerebro-vascular or brain or cerebral or cerebrum) adj2 (accident$ or lesion$ or attack$ or insult$ or insufficienc$ or arrest$ or fail$ or injur$)) or stroke or apoplex$).ti,ab,kw. | 660,307 |
| 22 | heart failure/ | 303,307 |
| 23 | ((heart or cardiac or cardial or myocardial) adj2 (fail$ or decompensation or incompeten$ or insuffien$ or stand still)).ti,ab,kw. | 373,533 |
| 24 | hypertension/ | 731,355 |
| 25 | ((hypertensive adj1 (disease* or effect* or response*)) or hypertension or high blood pressure).ti,ab,kw. | 730,378 |
| 26 | chronic kidney failure/ | 145,478 |
| 27 | ((Chronic adj (kidney or renal) adj (failure$ or disease$ or insufficienc$)) or chronic nephropath$ or ckd or esrd).ti,ab,kw. | 205,537 |
| 28 | metabolic syndrome X/ | 101,445 |
| 29 | ((insulin resistance or metabolic) adj2 resistance).ti,ab,kw. | 140,798 |
| 30 | dyslipidemia/ | 98,098 |
| 31 | (dyslip?emia$ or dyslipid?emia$ or dys-lip?emia$ or dys-lipid?emia$).ti,ab,kw. | 80,206 |
| 32 | non insulin dependent diabetes mellitus/ | 332,838 |
| 33 | (T2dm or TiiDM or Tii-DM or "type 2 DM" or type-ii-DM or DM2 or DM-2 or "T2-DM" or NIDDM or (diabet$ adj3 ("typ$ 2" or "typ$ ii" or type2 or typeii or "typ$ two"))).ti,ab,kw. | 309,966 |
| 34 | (noninsulin$ depend$ or non-insulin$ depend).ti,ab,kw. | 1851 |
| 35 | obesity/ | 532,777 |
| 36 | (obese or obesity or adipose tissue hyperplasia or adipositas or adiposity or excess body weight or corpulen$ or fat overload syndrome$ or obesitas or overweight or over-weight).ti,ab,kw. | 617,269 |
| 37 | bone disease/ | 28,462 |
| 38 | ((Bone or skeletal or skeleton) adj (disease$ or disorder$)).ti,ab,kw. | 33,345 |
| 39 | biological marker/ | 442,616 |
| 40 | (Biological marker$ or biomarker$ or bio marker$).ti,ab,kw. | 636,368 |
| 41 | Health services accessibility/ | 70,938 |
| 42 | (access$ adj2 (medication$ or healthcare or care or health service$ or medicine$ or therap$ or treatment$ or program$)).ti,ab,kw. | 78,365 |
| 43 | "patient acceptance of health care"/ or patient compliance/ or medication adherence/ | 237,111 |
| 44 | ((therap$ or treatment$ or medication$ or medicine$ or patient$ or client$ or drug$) adj2 (adher$ or compl$ or noncompl$ or nonadher$ or persisten$ or participat$ or collaborat$)).ti,ab,kw. | 714,972 |
| 45 | Patient satisfaction/ or patient acceptance of health care/ | 230,289 |
| 46 | ((accept$ or nonaccept$) adj2 (health care or healthcare)).ti,ab,kw. | 948 |
| 47 | (((patient$ or treatment$) adj2 satisf$) or desire$ or view$).ti,ab,kw. | 983,217 |
| 48 | or/8-47 | 8,961,786 |
| 49 | (link$ or associat$ or mediat$ or predict$ or determinant$ or correlat$).ti,ab,kw. /freq=3 | 4,070,509 |
| 50 | dependent variable/ | 17,161 |
| 51 | or/49-50 | 4,078,664 |
| 52 | 7 and 48 and 51 | 2162 |
| 53 | (editorial or letter or comment or note).pt. | 3,022,249 |
| 54 | (conference abstract or conference paper).pt. | 5,533,395 |
| 55 | 52 not (53 or 54) | 1679 |
| 56 | limit 54 to yr="2020 -Current" | 940,436 |
| 57 | 52 and 56 | 116 |
| 58 | 55 or 57 | 1795 |
