# Supplementary material for: The burden of HIV-related stigma on clinical and quality of life outcomes: results from a systematic literature review
Source: Health Psychol Behav Med. 2026 Jul 28;14(1):2672790. doi: 10.1080/21642850.2026.2672790 (PMC13421116; doi:10.1080/21642850.2026.2672790)
Supplement: Supplemental Table 2.docx [file RHPB_A_2672790_SM2254.docx]

| **Supplemental Table 2.** OVID MEDLINE^®^ and Embase^®^ Search Strategies for Identifying Validated Patient-Reported Outcome Measures Used to Evaluate HIV-Related Stigma Levels Experienced by People With HIV (Date of Search: May 30, 2023)^a,b^ | | |
| --- | --- | --- |
| **Search strategy number** | **Search terms (MEDLINE^®^**) | **Number of records** |
| **Population experiencing HIV-related stigma** | | |
| 1 | HIV/ or HIV Infections/ | 234,019 |
| 2 | ((human immunodeficiency adj2 virus$) or (human immun? deficiency adj2 virus$) or acquired immun? deficiency syndrome virus$ or acquired immunodeficiency syndrome virus$ or (aids associated adj (lentivirus$ or retrovirus$ or virus$)) or aids related virus$ or aids virus$ or HIV$ or human t cell lymphotropic virus type iii or immunodeficiency associated virus$ or lav or PLHIV or ALHIV or PLWHA or (lymphadenopathy associated adj2 (retrovirus$ or virus$))).ti,ab,kf. /freq=3 | 222,041 |
| 3 | or/1-2 | 311,990 |
| 4 | Social Stigma/ or Prejudice/ or Perception/ or Taboo/ or Attitude/ or Social Isolation/ | 146,710 |
| 5 | (Stigma$ or ostraci$ or selfdiscriminat$ or discriminat$ or "fear of outing" or "fear of coming out" or ((perceived or actual or fear or felt or anticipated) adj7 (judg$ or prejudice$)) or "not accept$" or non accept$ or nonaccept$ or unaccept$ or un accept$ or victimi?ation or psychosocial factor$ or psycho-social factor$ or social alienat$ or marginal$).ti,ab,kf. | 516,438 |
| 6 | or/4-5 | 639,994 |
| **Patient-reported outcomes** | | |
| 7 | "Weights and Measures"/ or "Surveys and Questionnaires"/ | 562,803 |
| 8 | (tool$ or scale$ or metric$ or score$ or index or instrument$ or question$ or interview$ or measure$ or assess$ or survey).ti,ab,kf. /freq=3 | 2,844,389 |
| 9 | or/7-8 | 3,089,561 |
| 10 | 3 and 6 and 9 | 5040 |
| 11 | Patient Reported Outcome Measures/ | 13,368 |
| 12 | (("quality of life" or wellbeing or well-being or QoL or HRQoL or HRQL) adj5 (tool$ or questionnaire$ or scale$ or instrument$ or index or indices or measure$ or profile$ or assess$)).ti,ab. | 115,992 |
| 13 | ((patient$ or self) adj2 (report$ or apprais$) adj2 (rate$ or rating$ or response$ or evaluat$ or outcome$ or assess$ or measure$)).ti,ab. | 79,739 |
| 14 | ((function$ or health) adj2 status adj2 report$).ti,ab. | 3267 |
| 15 | (screen$ adj2 (tool$ or questionnaire$ or instrument$)).ti,ab. | 44,942 |
| 16 | (PROM$1 or PRO$1).ti,ab,kf. | 304,296 |
| 17 | or/11-16 | 529,151 |
| 18 | 3 and 6 and 17 | 457 |
| 19 | 10 or 18 | 5161 |
| **Study design** | | |
| 20 | (randomized controlled trial or controlled clinical trial).pt. | 683,784 |
| 21 | (randomized or placebo or randomly).ab. | 1,063,045 |
| 22 | clinical trials as topic.sh. | 200,983 |
| 23 | trial.ti. | 285,902 |
| 24 | or/20-23 | 1,525,130 |
| 25 | exp animals/ not humans.sh. | 5,124,303 |
| 26 | 24 not 25 | 1,403,707 |
| 27 | Epidemiologic studies/ | 9327 |
| 28 | exp case control studies/ | 1,417,503 |
| 29 | exp cohort studies/ | 2,483,750 |
| 30 | Case control.tw. | 152,994 |
| 31 | (cohort adj (study or studies)).tw. | 312,342 |
| 32 | Cohort analy$.tw. | 11,685 |
| 33 | (Follow up adj (study or studies)).tw. | 56,041 |
| 34 | (observational adj (study or studies)).tw. | 159,171 |
| 35 | Longitudinal.tw. | 319,597 |
| 36 | Retrospective.tw. | 736,891 |
| 37 | Cross sectional.tw. | 507,004 |
| 38 | Cross-sectional studies/ | 467,100 |
| 39 | or/27-38 | 3,768,609 |
| 40 | 26 or 39 | 4,848,454 |
| 41 | 19 and 40 | 2406 |
| **Named tools** | | |
| 42 | "AIDS attitude and conservative views scale$".ti,ab,kf. | 0 |
| 43 | (HIV$ Provider Stigma Inventor$ or HAPSI).ti,ab,kf. | 4 |
| 44 | (HIV Stigma Instrument$ or HASI).ti,ab,kf. | 140 |
| 45 | AIDS attitude scale$.ti,ab,kf. | 24 |
| 46 | attitude$ toward$ women with HIV$ scale$.ti,ab,kf. | 1 |
| 47 | nurse$ attitude$ about AIDS scale$.ti,ab,kf. | 1 |
| 48 | AIDS victim blam$ scale$.ti,ab,kf. | 1 |
| 49 | Feeling thermometer toward$ PLWA.ti,ab,kf. | 0 |
| 50 | expressed HIV$ related scale$.ti,ab,kf. | 0 |
| 51 | AIDS related stigma scale$.ti,ab,kf. | 26 |
| 52 | AIDS stigma scale$.ti,ab,kf. | 20 |
| 53 | or/42-52 | 212 |
| 54 | 41 or 53 | 2595 |
| 55 | (letter or comment or editorial).pt. | 2,161,472 |
| 56 | 54 not 55 | 2589 |
| **Search strategy number** | **Search terms (Embase^®^)** | **Number of records** |
| **Population experiencing HIV-related stigma** | | |
| 1 | *Human immunodeficiency virus/ or *Human immunodeficiency virus infection/ or *Human immunodeficiency virus infected patient/ | 264,339 |
| 2 | ((human immunodeficiency adj2 virus$) or (human immun? deficiency adj2 virus$) or acquired immun? deficiency syndrome virus$ or acquired immunodeficiency syndrome virus$ or (aids associated adj (lentivirus$ or retrovirus$ or virus$)) or aids related virus$ or aids virus$ or HIV$ or human t cell lymphotropic virus type iii or immunodeficiency associated virus$ or lav or PLHIV or ALHIV or PLWHA or (lymphadenopathy associated adj2 (retrovirus$ or virus$))).ti,ab,kw. /freq=3 | 276,945 |
| 3 | or/1-2 | 373,445 |
| 4 | Stigma/ or social stigma/ or perception/ or psychological well-being/ or taboo/ or social attitude/ or social isolation/ or social exclusion/ | 258,083 |
| 5 | (Stigma$ or ostraci$ or selfdiscriminat$ or discriminat$ or "fear of outing" or "fear of coming out" or ((perceived or actual or fear or felt or anticipated) adj7 (judg$ or prejudice$)) or "not accept$" or non accept$ or nonaccept$ or unaccept$ or un accept$ or victimi?ation or psychosocial factor$ or psycho-social factor$ or social alienat$ or marginal$).ti,ab,kw. | 656,619 |
| 6 | or/4-5 | 875,304 |
| **Patient-reported outcomes** | | |
| 7 | measurement/ or questionnaire/ or interview/ | 1,195,557 |
| 8 | (tool$ or scale$ or metric$ or score$ or prevalence or index or instrument$ or question$ or interview$ or measure$ or assess$ or survey).ti,ab,kw. /freq=3 | 4,432,211 |
| 9 | or/7-8 | 4,915,258 |
| 10 | 3 and 6 and 9 | 8229 |
| 11 | patient-reported outcome/ | 53,053 |
| 12 | (("quality of life" or wellbeing or well-being or QoL or HRQoL or HRQL) adj5 (tool* or questionnaire* or scale* or instrument* or index or indices or measure* or profile* or assess*)).ti,ab. | 186,517 |
| 13 | ((patient* or self) adj2 (report* or apprais*) adj2 (rate* or rating* or response* or evaluat* or outcome* or assess* or measure*)).ti,ab. | 119,516 |
| 14 | ((function* or health) adj2 status adj2 report*).ti,ab. | 4418 |
| 15 | (screen* adj2 (tool* or questionnaire* or instrument*)).ti,ab. | 69,229 |
| 16 | (PROM$1 or PRO$1).ti,ab,kw. | 453,759 |
| 17 | or/11-16 | 804,008 |
| 18 | 3 and 6 and 17 | 730 |
| 19 | 10 or 18 | 8394 |
| **Study design** | | |
| 20 | Randomized controlled trial/ or Controlled clinical trial/ or randomization/ or intermethod comparison/ or double blind procedure/ or human experiment/ | 1,941,984 |
| 21 | (random$ or placebo or (open adj label) or parallel group$1 or ((double or single or doubly or singly) adj (blind or blinded or blindly)) or (crossover or cross over) or ((assign$ or match or matched or allocation) adj5 (alternate or group$1 or intervention$1 or patient$1 or subject$1 or participant$1)) or (assigned or allocated) or (controlled adj7 (study or design or trial)) or (volunteer or volunteers)).ti,ab. | 2,897,692 |
| 22 | (compare or compared or comparison or trial).ti. | 990,832 |
| 23 | ((evaluated or evaluate or evaluating or assessed or assess) and (compare or compared or comparing or comparison)).ab. | 2,766,233 |
| 24 | or/20-23 | 6,311,420 |
| 25 | (random$ adj sampl$ adj7 (cross section$ or questionnaire$1 or survey$ or database$1)).ti,ab. not (comparative study/ or controlled study/ or randomi?ed controlled.ti,ab. or randomly assigned.ti,ab.) | 9459 |
| 26 | Cross-sectional study/ not (randomized controlled trial/ or controlled clinical study/ or controlled study/ or randomi?ed controlled.ti,ab. or control group$1.ti,ab.) | 347,661 |
| 27 | (((case adj control$) and random$) not randomi?ed controlled).ti,ab. | 21,594 |
| 28 | (Systematic review not (trial or study)).ti. | 260,783 |
| 29 | (nonrandom$ not random$).ti,ab. | 18,928 |
| 30 | Random field$.ti,ab. | 2947 |
| 31 | (random cluster adj3 sampl$).ti,ab. | 1542 |
| 32 | (review.ab. and review.pt.) not trial.ti. | 1,117,570 |
| 33 | we searched.ab. and (review.ti. or review.pt.) | 49,788 |
| 34 | update review.ab. | 138 |
| 35 | (databases adj4 searched).ab. | 62,429 |
| 36 | (rat or rats or mouse or mice or swine or porcine or murine or sheep or lambs or pigs or piglets or rabbit or rabbits or cat or cats or dog or dogs or cattle or bovine or monkey or monkeys or trout or marmoset$1).ti. and animal experiment/ | 1,226,992 |
| 37 | Animal experiment/ not (human experiment/ or human/) | 2,577,203 |
| 38 | or/25-37 | 4,332,494 |
| 39 | 24 not 38 | 5,570,372 |
| 40 | Clinical study/ | 163,131 |
| 41 | Case control study/ | 206,184 |
| 42 | Family study/ | 25,770 |
| 43 | Longitudinal study/ | 193,700 |
| 44 | Retrospective study/ | 1,470,030 |
| 45 | Prospective study/ | 877,854 |
| 46 | Randomized controlled trials/ | 260,827 |
| 47 | 45 not 46 | 866,868 |
| 48 | Cohort analysis/ | 1,037,959 |
| 49 | (Cohort adj (study or studies)).mp. | 478,682 |
| 50 | (Case control adj (study or studies)).tw. | 169,114 |
| 51 | (follow up adj (study or studies)).tw. | 73,823 |
| 52 | (observational adj (study or studies)).tw. | 254,323 |
| 53 | (epidemiologic$ adj (study or studies)).tw. | 122,661 |
| 54 | (cross sectional adj (study or studies)).tw. | 338,643 |
| 55 | or/40-44,47-54 | 3,922,678 |
| 56 | 39 or 55 | 8,355,853 |
| 57 | 19 and 56 | 3531 |
| **Named tools** | | |
| 58 | (HIV$ Provider Stigma Inventor$ or HAPSI).ti,ab,kw. | 2 |
| 59 | (HIV Stigma Instrument$ or HASI).ti,ab,kw. | 93 |
| 60 | "AIDS attitude scale$".ti,ab,kw. | 31 |
| 61 | attitude$ toward$ women with HIV$ scale$.ti,ab,kw. | 1 |
| 62 | "AIDS attitude and conservative views scale$".ti,ab,kw. | 0 |
| 63 | nurse$ attitude$ about AIDS scale$.ti,ab,kw. | 1 |
| 64 | AIDS victim blam$ scale$.ti,ab,kw. | 1 |
| 65 | Feeling thermometer toward$ PLWA.ti,ab,kw. | 0 |
| 66 | expressed HIV$ related scale$.ti,ab,kw. | 0 |
| 67 | AIDS related stigma scale$.ti,ab,kw. | 28 |
| 68 | AIDS stigma scale$.ti,ab,kw. | 24 |
| 69 | or/58-68 | 176 |
| 70 | 57 or 69 | 3684 |
| 71 | (editorial or letter or comment or note).pt. | 3,023,285 |
| 72 | (conference abstract or conference paper).pt. | 5,533,404 |
| 73 | 70 not (71 or 72) | 2750 |
| 74 | limit 72 to yr="2020 -Current" | 940,444 |
| 75 | 70 and 74 | 255 |
| 76 | 73 or 75 | 3005 |
| ^a^Trials filter from: Lefebvre C, Glanville J, Briscoe S, Littlewood A, Marshall C, Metzendorf M-I, Noel-Storr A, Rader T, Shokraneh F, Thomas J, Wieland LS. Technical Supplement to Chapter 4: Searching for and selecting studies. In: Higgins JPT, Thomas J, Chandler J, Cumpston MS, Li T, Page MJ, Welch VA (eds). Cochrane Handbook for Systematic Reviews of Interventions Version 6.2 (updated February 2021). Cochrane, 2021. Available from: [www.training.cochrane.org/handbook](http://www.training.cochrane.org/handbook). ^b^Observational filter from: Scottish Intercollegiate Guidelines Network. Search filters [Internet]. [n.d.] [accessed 11.10.21]. Available from: <https://www.sign.ac.uk/what-we-do/methodology/search-filters/>. | | |
