# Supplementary material for: The burden of HIV-related stigma on clinical and quality of life outcomes: results from a systematic literature review
Source: Health Psychol Behav Med. 2026 Jul 28;14(1):2672790. doi: 10.1080/21642850.2026.2672790 (PMC13421116; doi:10.1080/21642850.2026.2672790)
Supplement: Supplemental Table 1.docx [file RHPB_A_2672790_SM2255.docx]

| **Supplemental Table 1.** OVID MEDLINE^®^ and Embase^®^ Search Strategies for Identifying Prevalence of HIV-Related Stigma Among People With HIV (Date of Search: May 30, 2023) | | |
| --- | --- | --- |
| **Search strategy number** | **Search terms (MEDLINE^®^**) | **Number of records** |
| **Population experiencing HIV-related stigma** | | |
| 1 | HIV/ or HIV Infections/ | 234,019 |
| 2 | ((human immunodeficiency adj2 virus$) or (human immun? deficiency adj2 virus$) or acquired immun? deficiency syndrome virus$ or acquired immunodeficiency syndrome virus$ or (aids associated adj (lentivirus$ or retrovirus$ or virus$)) or aids related virus$ or aids virus$ or HIV$ or human t cell lymphotropic virus type iii or immunodeficiency associated virus$ or lav or PLHIV or ALHIV or PLWHA or (lymphadenopathy associated adj2 (retrovirus$ or virus$))).ti,ab,kf. /freq=3 | 222,041 |
| 3 | or/1-2 | 311,990 |
| 4 | Social Stigma/ or Prejudice/ or Perception/ or Taboo/ or Attitude/ or Social Isolation/ | 146,710 |
| 5 | (Stigma$ or ostraci$ or selfdiscriminat$ or discriminat$ or "fear of outing" or "fear of coming out" or ((perceived or actual or fear or felt or anticipated) adj7 (judg$ or prejudice$)) or "not accept$" or non accept$ or nonaccept$ or unaccept$ or un accept$ or victimi?ation or psychosocial factor$ or psycho-social factor$ or social alienat$ or marginal$).ti,ab,kf. | 516,438 |
| 6 | or/4-5 | 639,994 |
| **Prevalence** | | |
| 7 | Incidence/ or Prevalence/ or Mortality/ or Demography/ or Epidemiology/ or Disease Progression/ or Morbidity/ | 933,770 |
| 8 | (occurrence$ or incidence$ or prevalence$ or episode$ or mortalit$ or morbidit$ or epidemiolog$ or demograph$).ti,ab. | 3,642,290 |
| 9 | or/7-8 | 3,981,174 |
| 10 | 3 and 6 and 9 | 5167 |
| 11 | limit 10 to yr="2020 -Current" | 1267 |
| 12 | (letter or comment or editorial).pt. | 2,161,472 |
| 13 | 11 not 12 | 1266 |
| **Search strategy number** | **Search terms (Embase^®^)** | **Number of records** |
| **Population experiencing HIV-related stigma** | | |
| 1 | *Human immunodeficiency virus/ or *Human immunodeficiency virus infection/ or *Human immunodeficiency virus infected patient/ | 264,339 |
| 2 | ((human immunodeficiency adj2 virus$) or (human immun? deficiency adj2 virus$) or acquired immun? deficiency syndrome virus$ or acquired immunodeficiency syndrome virus$ or (aids associated adj (lentivirus$ or retrovirus$ or virus$)) or aids related virus$ or aids virus$ or HIV$ or human t cell lymphotropic virus type iii or immunodeficiency associated virus$ or lav or PLHIV or ALHIV or PLWHA or (lymphadenopathy associated adj2 (retrovirus$ or virus$))).ti,ab,kw. /freq=3 | 276,945 |
| 3 | or/1-2 | 373,445 |
| 4 | Stigma/ or social stigma/ or perception/ or psychological well-being/ or taboo/ or social attitude/ or social isolation/ or social exclusion/ | 258,083 |
| 5 | (Stigma$ or ostraci$ or selfdiscriminat$ or discriminat$ or "fear of outing" or "fear of coming out" or ((perceived or actual or fear or felt or anticipated) adj7 (judg$ or prejudice$)) or "not accept$" or non accept$ or nonaccept$ or unaccept$ or un accept$ or victimi?ation or psychosocial factor$ or psycho-social factor$ or social alienat$ or marginal$).ti,ab,kw. | 656,619 |
| 6 | or/4-5 | 875,304 |
| **Prevalence** | | |
| 7 | incidence/ or standardized incidence ratio/ or Prevalence/ or standardized mortality ratio/ or demography/ or epidemiological data/ or mortality/ or disease progression/ or disease activity/ or morbidity/ | 2,944,236 |
| 8 | (occurrence$ or incidence$ or prevalence$ or episode$ or mortalit$ or morbidit$ or epidemiolog$ or demograph$).ti,ab. | 5,258,503 |
| 9 | or/7-8 | 6,036,124 |
| 10 | 3 and 6 and 9 | 6967 |
| 11 | limit 10 to yr="2020 -Current" | 1872 |
| 12 | (editorial or letter or comment or note).pt. | 3,023,285 |
| 13 | 11 not 12 | 1840 |
