# Supplementary material for: The burden of HIV-related stigma on clinical and quality of life outcomes: results from a systematic literature review
Source: Health Psychol Behav Med. 2026 Jul 28;14(1):2672790. doi: 10.1080/21642850.2026.2672790 (PMC13421116; doi:10.1080/21642850.2026.2672790)
Supplement: Supplemental Table 4.docx [file RHPB_A_2672790_SM2256.docx]

| **Supplemental Table 4.** OVID MEDLINE^®^ and Embase^®^ Search Strategies for Identifying the Impact of HIV-Related Stigma on Medication Use for Comorbid Conditions, Drug and Substance Use, and Related Healthcare Resource Utilization Costs Among People With HIV (Date of Search: May 25, 2023) | | |
| --- | --- | --- |
| **Search strategy number** | **Search terms (MEDLINE**^®^**)** | **Number of records** |
| **Population experiencing HIV-related stigma** | | |
| 1 | HIV/ or HIV Infections/ | 233,921 |
| 2 | ((human immunodeficiency adj2 virus$) or (human immun? deficiency adj2 virus$) or acquired immun? deficiency syndrome virus$ or acquired immunodeficiency syndrome virus$ or (aids associated adj (lentivirus$ or retrovirus$ or virus$)) or aids related virus$ or aids virus$ or HIV$ or human t cell lymphotropic virus type iii or immunodeficiency associated virus$ or lav or PLHIV or ALHIV or PLWHA or (lymphadenopathy associated adj2 (retrovirus$ or virus$))).ti,ab,kf. /freq=3 | 221,905 |
| 3 | or/1-2 | 311,823 |
| 4 | Social Stigma/ or Prejudice/ or Perception/ or Taboo/ or Attitude/ or Social Isolation/ | 146,677 |
| 5 | (Stigma$ or ostraci$ or selfdiscriminat$ or discriminat$ or "fear of outing" or "fear of coming out" or ((perceived or actual or fear or felt or anticipated) adj7 (judg$ or prejudice$)) or "not accept$" or non accept$ or nonaccept$ or unaccept$ or un accept$ or victimi?ation or psychosocial factor$ or psycho-social factor$ or social alienat$ or marginal$).ti,ab,kf. | 515,989 |
| 6 | or/4-5 | 639,528 |
| 7 | 3 and 6 | 17,213 |
| **Outcomes** | | |
| 8 | Comorbidity/ or Multimorbidity/ | 126,833 |
| 9 | (comorbid$ or co-morbid$ or multi-morbid$ or multimorbid$ or multidisease$ or multi-disease$ or ((multiple or co-existing) adj (disease$ or illness$ or condition$ or disorder$))).ti,ab,kf. | 270,256 |
| 10 | Depression/ or anxiety/ or inflammation/ | 422,139 |
| 11 | (depress$ or anxiet$ or anxious$ or inflammat$).ti,ab,kf. | 1,870,917 |
| 12 | Substance-Related Disorders/ | 105,283 |
| 13 | ((Substance or drug) adj2 (abuse$ or problem$ or overuse$ or disorder$)).ti,ab,kf. | 81,192 |
| 14 | or/8-13 | 2,321,748 |
| 15 | 7 and 14 | 2776 |
| **Healthcare resource use** | | |
| 16 | exp Employment/ or exp Work/ or Efficiency/ or Absenteeism/ or "Cost of Illness"/ or exp Cost Control/ or Budgets/ or Hospital Costs/ or Health Care Costs/ or "Length of Stay"/ | 387,780 |
| 17 | ((employment or employed or employee$ or unemployment or unemployed) adj3 (economic$ or cost or costs or costly or costing or price or prices or pricing or expenditure$)).ti,ab. | 3052 |
| 18 | (productivity adj3 (economic$ or cost or costs or costly or costing or price or prices or pricing or expenditure$)).ti,ab. | 4071 |
| 19 | ((long standing or longstanding or long term or longterm or permanent or employee$) adj2 (absence$ or absent$ or ill$ or sick$ or disab$)).ti,ab. | 13,124 |
| 20 | (cost$ adj2 (illness or disease$ or sickness$)).ti,ab. | 6047 |
| 21 | (burden$ adj2 (disease$ or illness or sickness$)).ti,ab. | 38,595 |
| 22 | ((social or societ$ or work$ or employe$ or business$ or communit$ or famil$ or carer$ or caregiver$) adj3 (burden$ or consequenc$ or impact$ or problem$ or productivity or sickness or impairment$)).ti,ab. | 128,162 |
| 23 | ((allowance or status or long-term or pension$ or benefit$) adj2 disab$).ti,ab. | 16,282 |
| 24 | ((unable or inability or incapacit$ or incapab$) adj3 work).ti,ab. | 2187 |
| 25 | budget$ impact$.ti,ab. | 2149 |
| 26 | budget$ implicat$.ti,ab. | 83 |
| 27 | (cost$ saving or cost$ savings or cost$ saved).ti,ab. | 23,536 |
| 28 | (cost$ adj2 contain$).ti,ab,ot. | 7237 |
| 29 | (cost$ adj2 audit$).ti,ab. | 158 |
| 30 | resource$ use$.ti,ab. | 12,976 |
| 31 | resource$ utili$.ti,ab. | 14,936 |
| 32 | resource$ usage.ti,ab. | 602 |
| 33 | (length adj2 stay$).ti,ab. | 78,153 |
| 34 | (hospital$ adj2 stay$).ti,ab. | 113,882 |
| 35 | (duration adj2 stay$).ti,ab. | 4617 |
| 36 | extended stay$.ti,ab. | 255 |
| 37 | prolonged stay$.ti,ab. | 1147 |
| 38 | ((hospitali?ation or hospitali?ed or hospital) adj3 (economic$ or cost or costs or costly or costing or price or prices or pricing or expenditure$ or budget$)).ti,ab. | 27,761 |
| 39 | (economic consequenc$ or cost consequenc$).ti,ab. | 5412 |
| 40 | or/16-39 | 722,915 |
| 41 | 15 and 40 | 209 |
| 42 | (letter or comment or editorial).pt. | 2,160,281 |
| 43 | 41 not 42 | 208 |
| **Search strategy number** | **Search terms (Embase^®^)** | **Number of records** |
| **Population experiencing HIV-related stigma** | | |
| 1 | *Human immunodeficiency virus/ or *Human immunodeficiency virus infection/ or *Human immunodeficiency virus infected patient/ | 264,312 |
| 2 | ((human immunodeficiency adj2 virus$) or (human immun? deficiency adj2 virus$) or acquired immun? deficiency syndrome virus$ or acquired immunodeficiency syndrome virus$ or (aids associated adj (lentivirus$ or retrovirus$ or virus$)) or aids related virus$ or aids virus$ or HIV$ or human t cell lymphotropic virus type iii or immunodeficiency associated virus$ or lav or PLHIV or ALHIV or PLWHA or (lymphadenopathy associated adj2 (retrovirus$ or virus$))).ti,ab,kw. /freq=3 | 276,910 |
| 3 | or/1-2 | 373,399 |
| 4 | Stigma/ or social stigma/ or perception/ or psychological well-being/ or taboo/ or social attitude/ or social isolation/ or social exclusion/ | 257,980 |
| 5 | (Stigma$ or ostraci$ or selfdiscriminat$ or discriminat$ or "fear of outing" or "fear of coming out" or ((perceived or actual or fear or felt or anticipated) adj7 (judg$ or prejudice$)) or "not accept$" or non accept$ or nonaccept$ or unaccept$ or un accept$ or victimi?ation or psychosocial factor$ or psycho-social factor$ or social alienat$ or marginal$).ti,ab,kw. | 656,484 |
| 6 | or/4-5 | 875,084 |
| 7 | 3 and 6 | 20,100 |
| **Outcomes** | | |
| 8 | Comorbidity/ | 385,402 |
| 9 | (comorbid$ or co-morbid$ or multi-morbid$ or multimorbid$ or multidisease$ or multi-disease$ or ((multiple or co-existing) adj (disease$ or illness$ or condition$ or disorder$))).ti,ab,kw. | 486,621 |
| 10 | Depression/ or anxiety/ or inflammation/ | 1,210,279 |
| 11 | (depress$ or anxiet$ or anxious$ or inflammat$).ti,ab,kw. | 2,659,924 |
| 12 | Substance abuse/ or drug abuse/ | 110,392 |
| 13 | ((Substance or drug) adj2 (abuse$ or problem$ or overuse$ or disorder$)).ti,ab,kw. | 105,885 |
| 14 | or/8-13 | 3,508,410 |
| 15 | 7 and 14 | 3605 |
| **Healthcare resource use** | | |
| 16 | exp employment/ or exp work/ or "cost of illness"/ or cost control/ or hospital cost/ or budget/ or health care cost/ or "length of stay"/ or health care utilization/ | 1,144,604 |
| 17 | ((employment or employed or employee$ or unemployment or unemployed) adj3 (economic$ or cost or costs or costly or costing or price or prices or pricing or expenditure$)).ti,ab. | 3850 |
| 18 | (productivity adj3 (economic$ or cost or costs or costly or costing or price or prices or pricing or expenditure$)).ti,ab. | 5833 |
| 19 | ((long standing or longstanding or long term or longterm or permanent or employee$) adj2 (absence$ or absent$ or ill$ or sick$ or disab$)).ti,ab. | 18,533 |
| 20 | (cost$ adj2 (illness or disease$ or sickness$)).ti,ab. | 9493 |
| 21 | (burden$ adj2 (disease$ or illness or sickness$)).ti,ab. | 60,777 |
| 22 | ((social or societ$ or work$ or employe$ or business$ or communit$ or famil$ or carer$ or caregiver$) adj3 (burden$ or consequenc$ or impact$ or problem$ or productivity or sickness or impairment$)).ti,ab. | 171,763 |
| 23 | ((allowance or status or long-term or pension$ or benefit$) adj2 disab$).ti,ab. | 26,828 |
| 24 | ((unable or inability or incapacit$ or incapab$) adj3 work).ti,ab. | 3255 |
| 25 | budget$ impact$.ti,ab. | 5867 |
| 26 | budget$ implicat$.ti,ab. | 126 |
| 27 | (cost$ saving or cost$ savings or cost$ saved).ti,ab. | 40,687 |
| 28 | (cost$ adj2 contain$).ti,ab. | 9,220 |
| 29 | (cost$ adj2 audit$).ti,ab. | 261 |
| 30 | resource$ use$.ti,ab. | 20,115 |
| 31 | resource$ utili$.ti,ab. | 28,239 |
| 32 | resource$ usage.ti,ab. | 873 |
| 33 | (length adj2 stay$).ti,ab. | 151,047 |
| 34 | (hospital$ adj2 stay$).ti,ab. | 192,220 |
| 35 | (duration adj2 stay$).ti,ab. | 7374 |
| 36 | extended stay$.ti,ab. | 397 |
| 37 | prolonged stay$.ti,ab. | 1896 |
| 38 | ((hospitali?ation or hospitali?ed or hospital) adj3 (economic$ or cost or costs or costly or costing or price or prices or pricing or expenditure$ or budget$)).ti,ab. | 45,895 |
| 39 | (economic consequenc$ or cost consequenc$).ti,ab. | 7263 |
| 40 | or/16-39 | 1,549,719 |
| 41 | 15 and 40 | 490 |
| 42 | (editorial or letter or comment or note).pt. | 3,022,249 |
| 43 | (conference abstract or conference paper).pt. | 5,533,395 |
| 44 | 41 not (42 or 43) | 379 |
| 45 | limit 43 to yr="2020 -Current" | 940,436 |
| 46 | 41 and 45 | 27 |
| 47 | 44 or 46 | 406 |
